# ORL@Cu‐MOF Boost Cuproptosis and Suppress Fatty Acid Metabolism for Cancer Lymph Node Metastasis Synergistic Therapy

**DOI:** 10.1002/advs.202502154

**Published:** 2025-06-23

**Authors:** Zi‐Zhan Li, Yi Liu, Kan Zhou, Lei‐Ming Cao, Guang‐Rui Wang, Jinmei Wu, Yi‐Fu Yu, Yao Xiao, Bing Liu, Qiuji Wu, Zhiyong Song, Lin‐Lin Bu

**Affiliations:** ^1^ State Key Laboratory of Oral & Maxillofacial Reconstruction and Regeneration Key Laboratory of Oral Biomedicine Ministry of Education Hubei Key Laboratory of Stomatology School & Hospital of Stomatology Wuhan University Wuhan 430079 China; ^2^ Department of Oral & Maxillofacial – Head Neck Oncology School & Hospital of Stomatology Wuhan University Wuhan 430079 China; ^3^ National Key Laboratory of Agricultural Microbiology College of Chemistry Huazhong Agricultural University Wuhan 430070 China; ^4^ Key Laboratory of Combinatorial Biosynthesis and Drug Discovery School of Pharmaceutical Sciences Wuhan University Wuhan 430071 China; ^5^ Department of Radiation and Medical Oncology Hubei Key Laboratory of Tumor Biological Behaviors Hubei Cancer Clinical Study Center Zhongnan Hospital of Wuhan University Wuhan 430071 China

**Keywords:** cuproptosis, immunotherapy, lipid metabolic reprogramming, lymph node metastasis, nanomedicine

## Abstract

Lymph node metastasis (LNM) is one of the significant characteristics of poor prognosis in oral squamous cell carcinoma (OSCC), strongly associated with high mortality rates. Immunotherapy has emerged as a crucial treatment modality for OSCC LNM, yet its limited response rate severely restricts its clinical application. Cuproptosis, a newly discovered immunogenic cell death (ICD), is often hindered by lipid metabolic reprogramming in tumor cells, which also contributes significantly to the lack of response to immunotherapy. Herein, a metal‐organic framework (MOF) nanodrug loaded with orlistat (ORL) is developed, designated as ORL@Cu‐MOF. This nanodrug is designed to respond and release under the high glutathione (GSH) stimulus of the tumor microenvironment (TME), thereby inducing cuproptosis and suppressing fatty acid metabolism in OSCC cells. In vivo, ORL@Cu‐MOF effectively triggers cuproptosis and inhibits fatty acid metabolism, exerting antitumor effects in mouse models and remodeling the TME. Furthermore, the combination of ORL@Cu‐MOF with programmed death receptor‐1 (αPD‐1) significantly enhances immunotherapy outcomes, transforming “cold tumors” into “hot tumors”. This study is the first to report the synergistic use of cuproptosis induction and fatty acid metabolism suppression in the treatment of metastatic cancer, offering novel insights for the care and management of LNM of OSCC.

## Graphical abstract

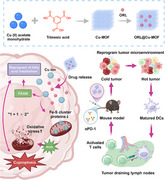

## 1 Introduction

Oral squamous cell carcinoma (OSCC) is the most common squamous cell carcinoma in the head and neck, and is the eighth most common malignancy worldwide.^[^ ^1^, ^2^ ^]^ Due to its high metastasis rate, the five‐year survival rate of patients with OSCC is less than 50%.^[^ ^3^ ^]^ OSCC mainly metastasizes to cervical lymph nodes (LN), and lymph node metastasis (LNM) is considered one of the most important markers of poor prognosis for patients with OSCC.^[^ ^4^, ^5^, ^6^ ^]^ Although the current main treatment for LNM is still surgery, maintaining the integrity of the LN as an important immune organ may be more beneficial to the prognosis of patients before the end of immunotherapy.^[^ ^7^, ^8^ ^]^ αPD‐1 (Anti‐programmed death receptor‐1) treatment can improve the prognosis of patients with OSCC and benefit patients with OSCC complicated with LNM, but its low clinical remission rate is an important reason limiting its use in the treatment of OSCC.^[^ ^9^, ^10^ ^]^ Moreover, metastatic cancers such as LNM exhibit stronger drug resistance to immunotherapy.^[^ ^6^ ^]^ Recent studies have shown that the synergistic effect of inducing immunogenic cell death (ICD) and αPD‐1 may be an important way to improve the response rate of patients with OSCC to αPD‐1.^[^ ^11^ ^]^

With the discovery of cuproptosis, cancer treatment strategies based on it have attracted significant attention.^[^ ^12^, ^13^ ^]^ Elesclomol (ES), as a copper ion carrier, transports extracellular copper into mitochondrial cells, ultimately leading to protein toxic stress and inducing cell death.^[^ ^14^ ^]^ Therefore, ES has emerged as an anticancer drug and entered clinical research. However, clinical studies have found that the therapeutic effect of ES is not satisfactory, as killing tumor cells solely through cuproptosis may not be sufficient to fully control tumor growth.^[^ ^15^, ^16^ ^]^ On one hand, tumor cells can resist oxidative stress by reprogramming lipid metabolism.^[^ ^17^ ^]^ Changes in fatty acid metabolism are important features of lipid metabolism reprogramming.^[^ ^18^ ^]^ Inhibiting lipid metabolism reprogramming while inducing oxidative stress will promote tumor cell death, such as inhibiting fatty acid synthase (FASN) to promote ferroptosis in hematological malignancies.^[^ ^19^ ^]^ Therefore, the combination of inducing cuproptosis and inhibiting lipid metabolism reprogramming may be an emerging therapeutic strategy for cancer. On the other hand, the biosafety of ES still needs to be verified through clinical trials, with reports of neurotoxicity studies emerging.^[^ ^20^ ^]^ There is an urgent need for safer drug delivery methods to enhance the value of cuproptosis in cancer treatment. In addition, as a form of ICD, cuproptosis can increase the expression of PD‐1.^[^ ^15^, ^21^ ^]^ Therefore, the combined use of cuproptosis inducers and existing αPD‐1 therapies may lead to more effective cancer treatment.

**Figure 1:**
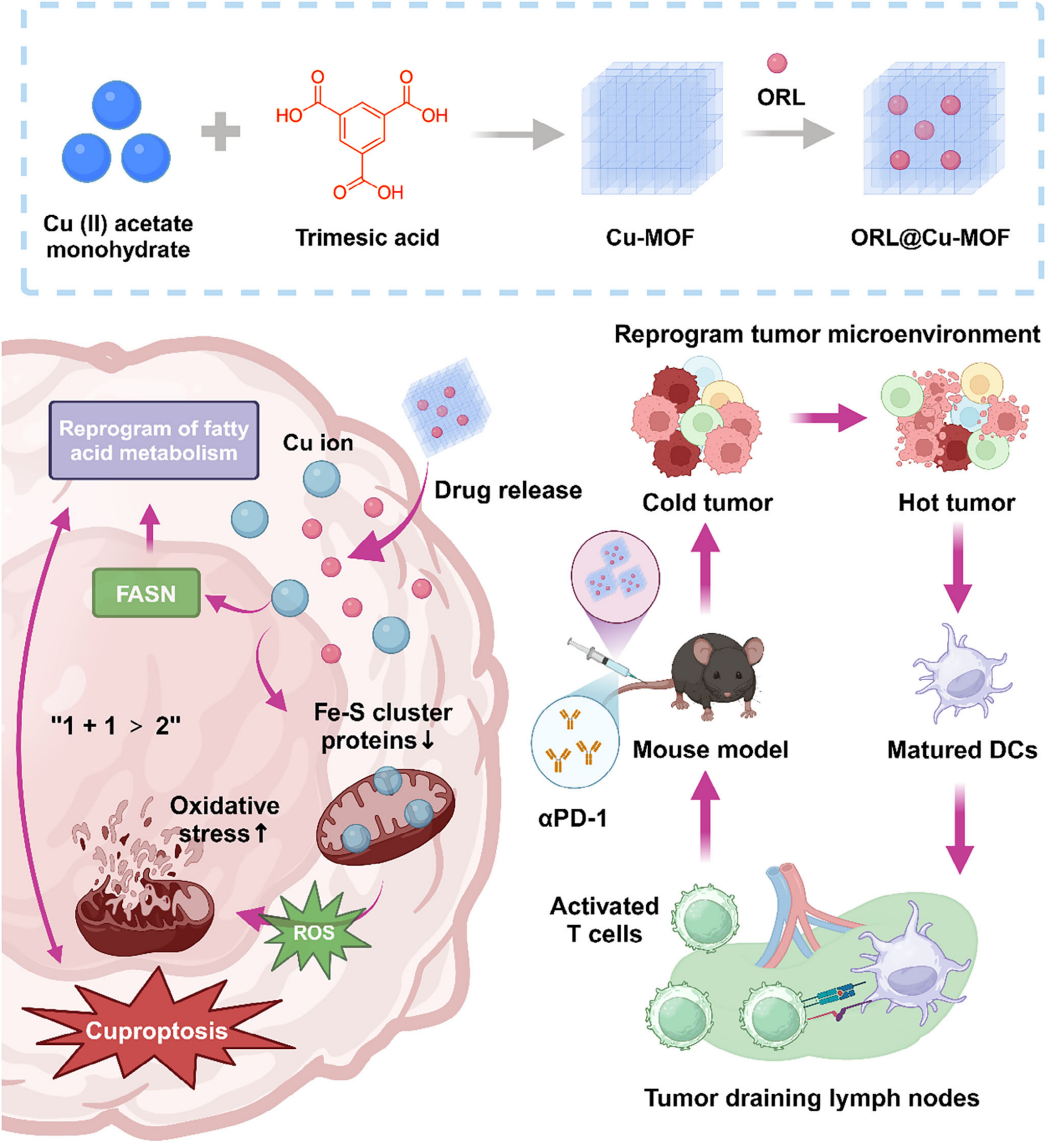
Schematic Illustration of ORL@Cu‐MOF boosts cuproptosis and suppresses fatty acid metabolism for cancer lymph node metastasis synergistic therapy. The fabrication of ORL@Cu‐MOF and the mechanism of synergistic therapy by cuproptosis, reprogram fatty acid metabolism, and anti‐PD‐1.

Cancer treatment based on cuproptosis is currently a focus of nanomedicine research.^[^ ^22^, ^23^ ^]^ The combination of cuproptosis with other anti‐tumor methods will significantly improve the treatment of OSCC and may generate new therapeutic ideas for the LNM of OSCC. This study designed a MOF structure that induces cuproptosis and can release Orlistat (ORL) in the high glutathione (GSH) environment of the tumor microenvironment (TME). ORL@Cu‐MOF is believed to selectively accumulate in the tumor and metastatic LN sites of OSCC mouse models. On the one hand, copper metal‐organic framework (MOF) releases Cu^2+^ and transports it to the mitochondria of tumor cells, inducing the production of reactive oxygen species (ROS), morphological and structural changes in mitochondria and endoplasmic reticulum, and inducing cuproptosis of OSCC cells. At the same time, ORL is released into the TME, inhibiting the expression of FASN in tumor cells and inhibiting the uptake of lipids and the formation of lipid droplets in OSCC cells, thereby inhibiting the reprogramming of lipid metabolism in OSCC, and this process promotes copper death of OSCC cells, resulting in a synergistic therapeutic effect. In animal experiments, by constructing a mouse model of footpad injection with popliteal/inguinal LNM, ORL@Cu‐MOF effectively inhibited the tumor progression of primary tumors and LN metastases. In addition, the increase of intracellular Cu^2+^ will significantly increase the expression of PD‐1 on the surface of tumor cells.^[^ ^9^, ^15^ ^]^ thus laying the groundwork for the combined use of ORL@Cu‐MOF and αPD‐1. On the other hand, ORL@Cu‐MOF can promote the maturation of dendritic cells (DC), improve the infiltration of CD4/CD8+ T cells within the TME, thereby converting “cold tumors” into “hot tumors”. In summary, the development of ORL@Cu‐MOF will provide a new therapeutic idea for the LNM of OSCC and can effectively improve the therapeutic effect of αPD‐1 on the LNM of OSCC, with broad clinical application prospects (**Figure** **1**).

## 2 Results and Discussion

### 2.1 Correlation Between Cuproptosis‐Related Genes, Fatty Acid Metabolism, and PD‐1 Expression in OSCC

DLAT, LIAS, and FDX1 are key genes involved in cuproptosis.^[^ ^24^ ^]^ Using the GEO database, we analyzed the expression levels of these genes in OSCC patients. The results revealed significantly higher expressions of DLAT, LIAS, and FDX1 in LNM+ OSCC patients compared to LNM‐ OSCC patients, indicating their potential involvement in the malignant progression of OSCC (Figure S1, Supporting Information). Furthermore, we explored the relationship between DLAT expression and the survival outcomes of OSCC patients. We found that OSCC patients with low DLAT expression exhibited longer disease‐free survival periods (Figure S2, Supporting Information).

However, targeted cancer therapy against DLAT remains challenging due to its limited therapeutic efficacy. Although targeting DLAT can induce ROS generation, it does not consistently demonstrate satisfactory tumoricidal effects.^[^ ^25^ ^]^ Lipid metabolism reprogramming is a crucial factor in tumor cell resistance to ROS stress.^[^ ^26^ ^]^ To investigate the potential synergistic effect of inducing cuproptosis and inhibiting fatty acid metabolism, we analyzed the expression correlation between DLAT, a key gene in cuproptosis, and FASN, the gene encoding a rate‐limiting enzyme in fatty acid metabolism. Our results showed a positive correlation between DLAT and FASN expressions (Figure S3, Supporting Information). We further analyzed the expression levels of FASN in LNM+ OSCC patients and LNM‐ OSCC patients. Consistently, FASN expression was significantly higher in OSCC tissues compared to adjacent tissues (Figure S4A, Supporting Information). Moreover, OSCC patients with high FASN expression exhibited shorter disease‐free survival periods (Figure S4B, Supporting Information). These findings suggest that targeting FASN may be an effective therapeutic strategy for OSCC and may synergize with inducing cuproptosis, laying an important foundation for further research in this area.

Additionally, we examined the correlation between DLAT and PD‐1 expressions. Interestingly, we found a positive correlation between the expression levels of DLAT and PD‐1 in OSCC tumor tissues (Figure S5, Supporting Information). These results imply a close relationship between the expression of cuproptosis‐related genes, fatty acid metabolism genes, and PD‐1, which may have implications for understanding the immune response and developing immunotherapy strategies in OSCC. Collectively, our findings provide valuable insights into the complex interactions between cuproptosis, fatty acid metabolism, and PD‐1 expression in OSCC.

### 2.2 Synthesis and Characterization of ORL@Cu‐MOF

**Figure 2:**
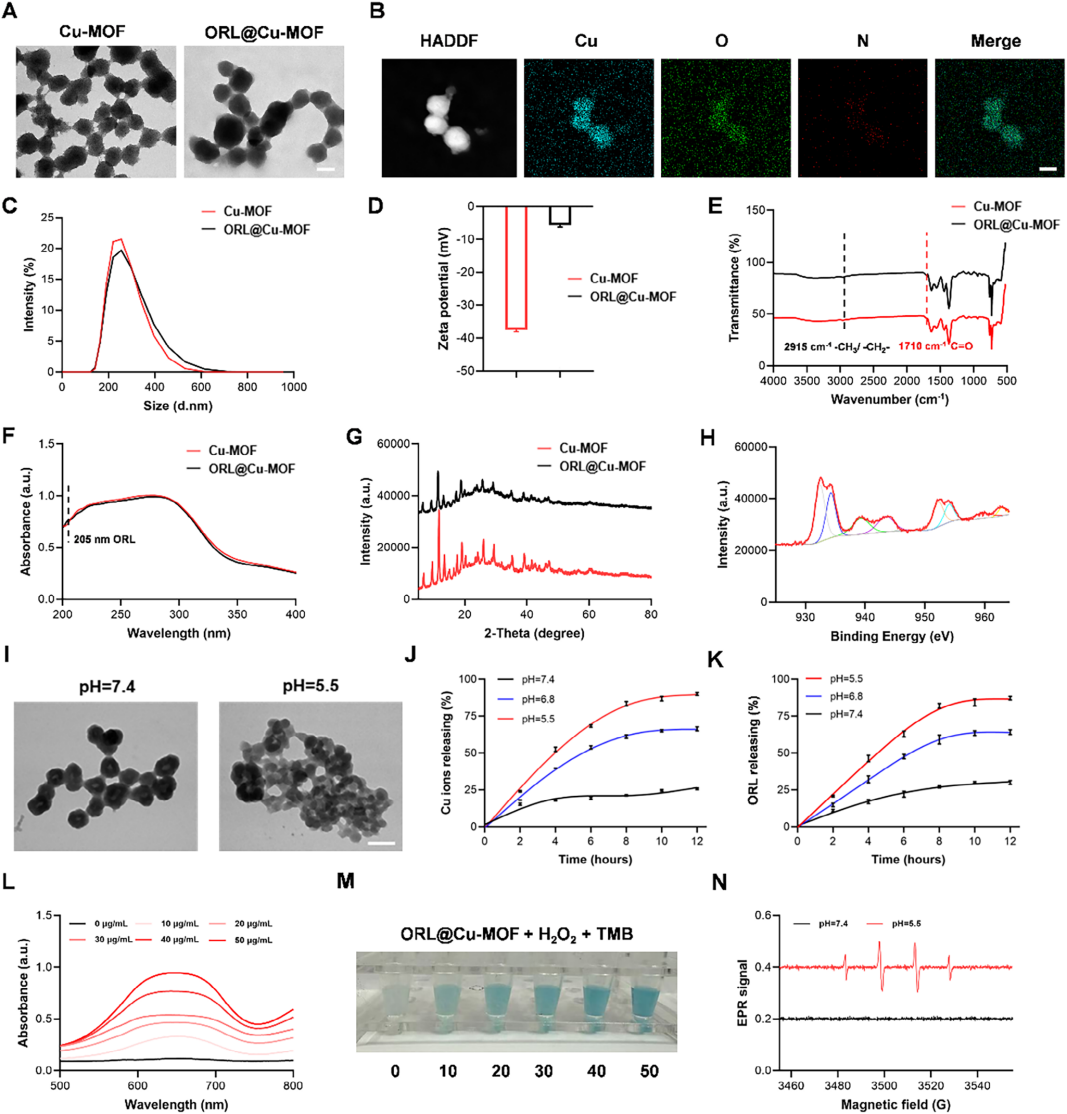
Synthesis and Characterization of ORL@Cu‐MOF. A) Transmission Electron Microscopy (TEM) image of ORL@Cu‐MOF; Scale bar, 200 nm. B) TEM elemental mapping of ORL@Cu‐MOF; Scale bar, 200 nm. C) Particle size distribution histogram. D) Zeta potential measurements. E) Fourier Transform Infrared (FTIR) absorption spectrum. F) UV–vis absorption spectrum. G) X‐ray Diffraction (XRD) pattern. H) X‐ray Photoelectron Spectroscopy (XPS) analysis for the Mn element. I) TEM images of ORL@Cu‐MOF in solutions with different pH values; scale bar, 200 nm. J) Cu ion release from ORL@Cu‐MOF in solutions with different pH values. K) ORL release from ORL@Cu‐MOF in solutions with different pH values. L,M) UV–vis absorption spectrum and colorimetric images of TMB reaction. N) Electron Paramagnetic Resonance (EPR) radical detection of ORL@Cu‐MOF in solutions with different pH values.

The Cu‐MOF was synthesized by referring to and improving upon the method described by previous studies,^[^ ^27^ ^]^ resulting in a uniform morphology. As shown in **Figure** **2**A, TEM images reveal that the Cu‐MOF exhibits a spherical structure with a size of 100 ± 15 nm and good dispersion (PDI = 0.025). Through electrostatic adsorption, ORL was loaded into the pore structure of Cu‐MOF. ORL@Cu‐MOF maintains a spherical shape and good dispersion (PDI = 0.081). To further confirm the successful synthesis of ORL@Cu‐MOF and drug loading, high‐angle annular dark‐field scanning transmission electron microscopy (HAADF‐STEM) and elemental mapping were conducted. As illustrated in Figure 2B, the N element from ORL and Cu, O elements show significant colocalization, indicating successful drug loading. Dynamic light scattering (DLS) studies (Figure 2C) show that Cu‐MOF has a particle size of ≈221 nm, while ORL@Cu‐MOF has a particle size of ≈228 nm. The Zeta potential measurements (Figure 2D) indicate that Cu‐MOF has an initial potential of −37.5 mV, which significantly increases to −5.53 mV upon ORL loading, providing preliminary evidence for successful ORL incorporation. Subsequently, UV–vis absorption spectroscopy and Fourier transform infrared (FT‐IR) spectroscopy were used to analyze the functional groups of ORL@Cu‐MOF and the UV absorption peak of ORL. As shown in Figure 2E,F, a distinct UV absorption peak of ORL is observed at 205 nm, and the FT‐IR spectrum exhibits characteristic peaks of CH_3−_, ─CH_2−_, and C═O from ORL at 2950 and 1710 cm^−1^, confirming the synthesis of ORL@Cu‐MOF and the loading of ORL. Next, to investigate whether the crystal structure of Cu‐MOF changes before and after drug loading, X‐ray diffraction (XRD) analysis was performed. As shown in Figure 2G, the positions of the XRD characteristic peaks of ORL@Cu‐MOF and Cu‐MOF remain unchanged, indicating that ORL loading does not affect the crystal structure of Cu‐MOF. Additionally, XPS valence state analysis of ORL@Cu‐MOF (Figure 2H) reveals distinct Cu valence state peaks. In conclusion, Cu‐MOF was successfully synthesized, and its porous properties were utilized to achieve the loading of the ORL.

### 2.3 Determination of Drug Release Ability of ORL@Cu‐MOF

The interior of tumor cells exhibits an acidic environment. To confirm the acid‐responsive drug release capability of ORL@Cu‐MOF, as illustrated in Figure 2I, TEM images reveal that at pH = 7.4, the nanostructure of ORL@Cu‐MOF remains intact without significant collapse, maintaining its spherical shape. As the pH environment changes to pH = 5.5, ORL@Cu‐MOF undergoes notable degradation, with alterations in its spherical morphology and a reduction in particle size. Subsequently, to verify that an acidic environment facilitates the release of ORL and Cu ions, tests were conducted under conditions of pH = 7.4 and pH = 5.5. As shown in Figure 2J,K, HPLC analysis of the released ORL indicates that at pH = 5.5, the release of ORL reaches 87.21%, at pH 6.8, with a release rate of 64.10%, whereas at pH = 7.4, the release capacity is only 30%. demonstrating that an acidic environment promotes the release of ORL. Cu ions exhibit a similar phenomenon, with a release capacity of 90.02% at pH = 5.5, whereas at pH 6.8, Cu ions still demonstrated significant release capacity, with a release rate of 66.36% and only 25.74% at pH = 7.4. Combining these findings confirms that ORL@Cu‐MOF possesses excellent acid‐responsive properties, enhancing the release of ORL and Cu ions. Furthermore, the interior of tumor cells is characterized by a high hydrogen peroxide environment. With the release of Cu ions, this promotes the decomposition of intracellular hydrogen peroxide, generating a Fenton reaction effect. We assessed the stability of different ORL@Cu‐MOF in PBS, FBS, and DMEM+FBS. The results showed that there were no significant changes in nanoparticle size and stability over time. Even after prolonged incubation, the nanoparticle size remained stable at ≈230 nm, indicating that ORL@Cu‐MOF exhibits excellent stability in these media (Figure S6, Supporting Information). To validate this function, as shown in Figure 2L, in vitro testing of the peroxidase (POD) enzyme activity of ORL@Cu‐MOF reveals that as the concentration of ORL@Cu‐MOF increases, the degree of TMB oxidation also increases, resulting in more pronounced POD enzyme activity. Finally, EPR was utilized to detect radicals associated with POD enzyme activity. As shown in Figure 2M, ORL@Cu‐MOF generates a significant amount of ·OH radicals in an acidic environment, exhibiting excellent POD enzyme activity. In summary, ORL@Cu‐MOF demonstrates a robust ability to respond to the acidic and high hydrogen peroxide environment of tumor cells, with notable drug release capacity and POD enzyme activity.

### 2.4 In Vitro Antitumor Activity of ORL@Cu‐MOF

**Figure 3:**
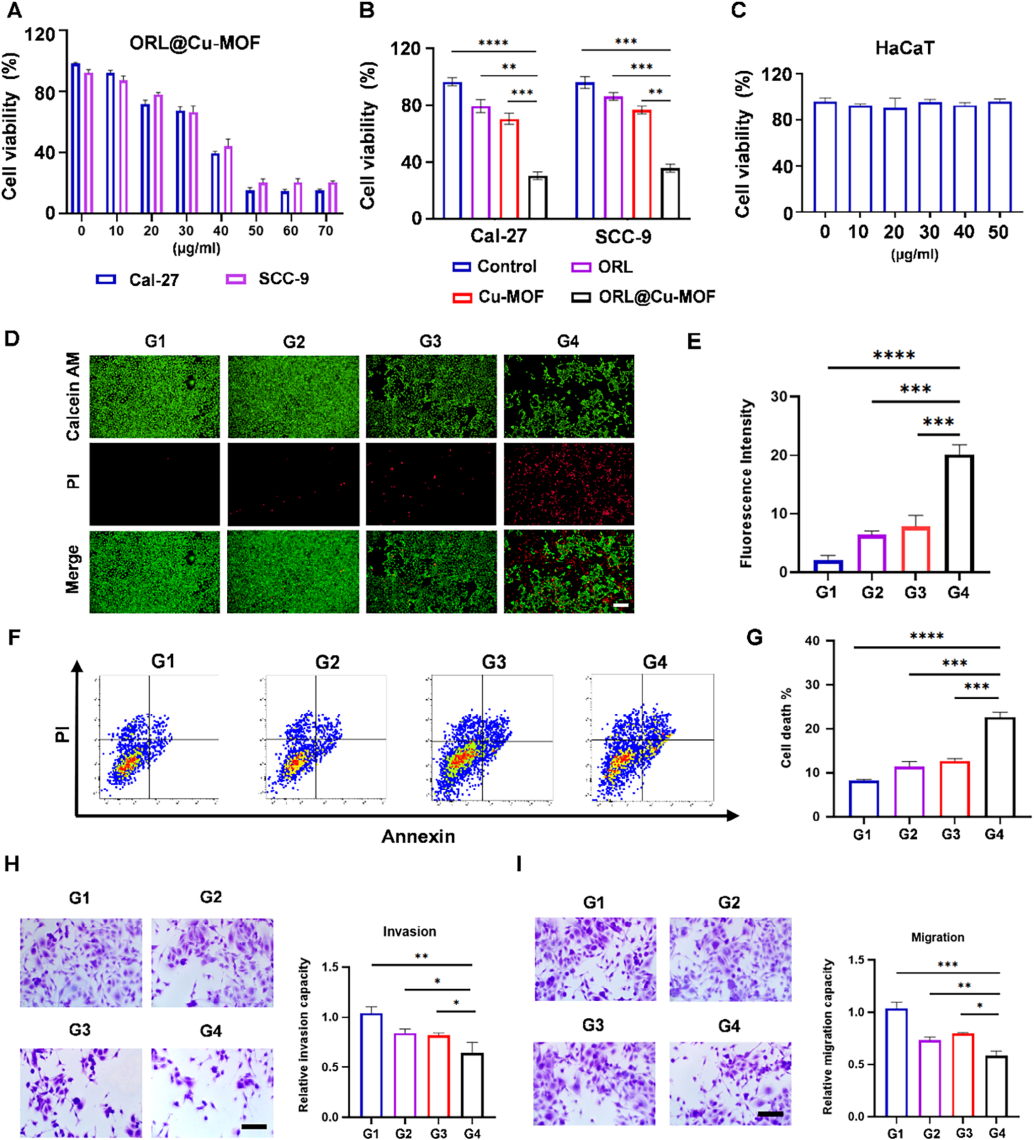
Antitumor Activity of ORL@Cu‐MOF in vitro. A) Impact of ORL@Cu‐MOF concentrations on the viability of Cal‐27 and SCC‐9 cells. B) Cellular viability of Cal‐27 and SCC‐9 cells following various treatments. C) Effect of ORL@Cu‐MOF concentrations on the viability of HaCaT cells. D) Representative CLSM images and E) semi‐quantification of Cal‐27 cells under various treatments, stained with calcein‐AM (green, viable cells) and PI (red, dead cells); Scale bar, 100 µm. F) Representative flow cytometry (FCM) profiles and G) semi‐quantification of the apoptotic rate of Cal‐27 cells after various treatments using FCM; Scale bar, 100 µm. H) Transwell images and semi‐quantification of the relative invasion capacity of Cal‐27 cells after various treatments; Scale bar, 100 µm. I) Transwell images and semi‐quantification of the relative migration capacity of Cal‐27 cells after various treatments; Scale bar, 100 µm. G1: Control; G2: ORL; G3: Cu‐MOF; G4: ORL@Cu‐MOF. Data are represented as mean ± SD, *n* = 4. One‐way ANOVA with Tukey's correction for statistical significance. ^*^ *p* < 0.05, ^**^ *p* < 0.01, ^***^ *p* < 0.001, ^****^ *p* < 0.0001.

To assess the antitumor activity of ORL@Cu‐MOF, Cal‐27 cells were treated with varying concentrations of the compound. As the concentration of ORL@Cu‐MOF increased, a more potent cytotoxic effect on tumor cells was observed (**Figure** **3**A). Consequently, a concentration of 50 µg mL^−1^ was selected for subsequent experiments. To validate the antitumor effect of different components and further confirm the activity of ORL@Cu‐MOF, the antitumor effects of ORL, Cu‐MOF, and ORL@Cu‐MOF were evaluated in two distinct OSCC cell lines, Cal‐27 and SCC‐9. Similar phenomena were observed in both cell lines. Although ORL has been considered a potential therapeutic agent for OSCC, its antitumor effect was not significant. Similarly, Cu‐MOF did not exhibit satisfactory antitumor activity. In contrast, ORL@Cu‐MOF demonstrated strong antitumor activity (Figure 3B). Importantly, the antitumor activity of ORL@Cu‐MOF was significantly higher than that of ORL and Cu‐MOF at the same concentration, indicating a potential synergistic effect between the two anticancer agents. Given that ORL@Cu‐MOF functions in a GSH‐responsive manner, and high GSH levels are a hallmark of the TME, we evaluated the potential cytotoxicity of different concentrations of ORL@Cu‐MOF in HaCaT cells, which are generally considered as a cell line represent normal oral mucosal epithelial cells. The results revealed that even at effective antitumor concentrations (50 µg mL^−1^), ORL@Cu‐MOF did not exhibit significant cytotoxicity against normal cells (Figure 3C), providing a foundation for the biosafety of ORL@Cu‐MOF.

Subsequently, live/dead staining of Cal‐27 cells was performed to more accurately mimic drug treatment effects, enabling faster and more cost‐effective preclinical drug testing. The results demonstrated that ORL@Cu‐MOF intervention resulted in the highest number of dead cells (red) (Figure 3D,E). Flow cytometry results further supported these findings, as the apoptotic rate of Cal‐27 cells treated with different drugs was explored using Annexin V‐FITC and propidium iodide (PI) double staining. The results indicated that the apoptotic proportion of cells treated with ORL@Cu‐MOF was 1.8 times higher than that of ORL and 1.3 times higher than that of Cu‐MOF (Figure 3F,G). In addition, we employed a Transwell chamber assay to assess alterations in the invasive and migratory capacities of Cal‐27 cells under different treatment conditions. Our findings demonstrated that ORL@Cu‐MOF exhibited significantly superior inhibitory effects on both cellular invasion and migration compared to ORL or Cu‐MOF alone (Figure 3H,I). Collectively, these findings confirm the good antitumor activity of ORL@Cu‐MOF *in* *vitro*, which is significantly stronger than the individual effects of ORL and Cu‐MOF.

### 2.5 ORL@Cu‐MOF can Induce Cuproptosis and Suppress Fatty Acid Metabolism

**Figure 4:**
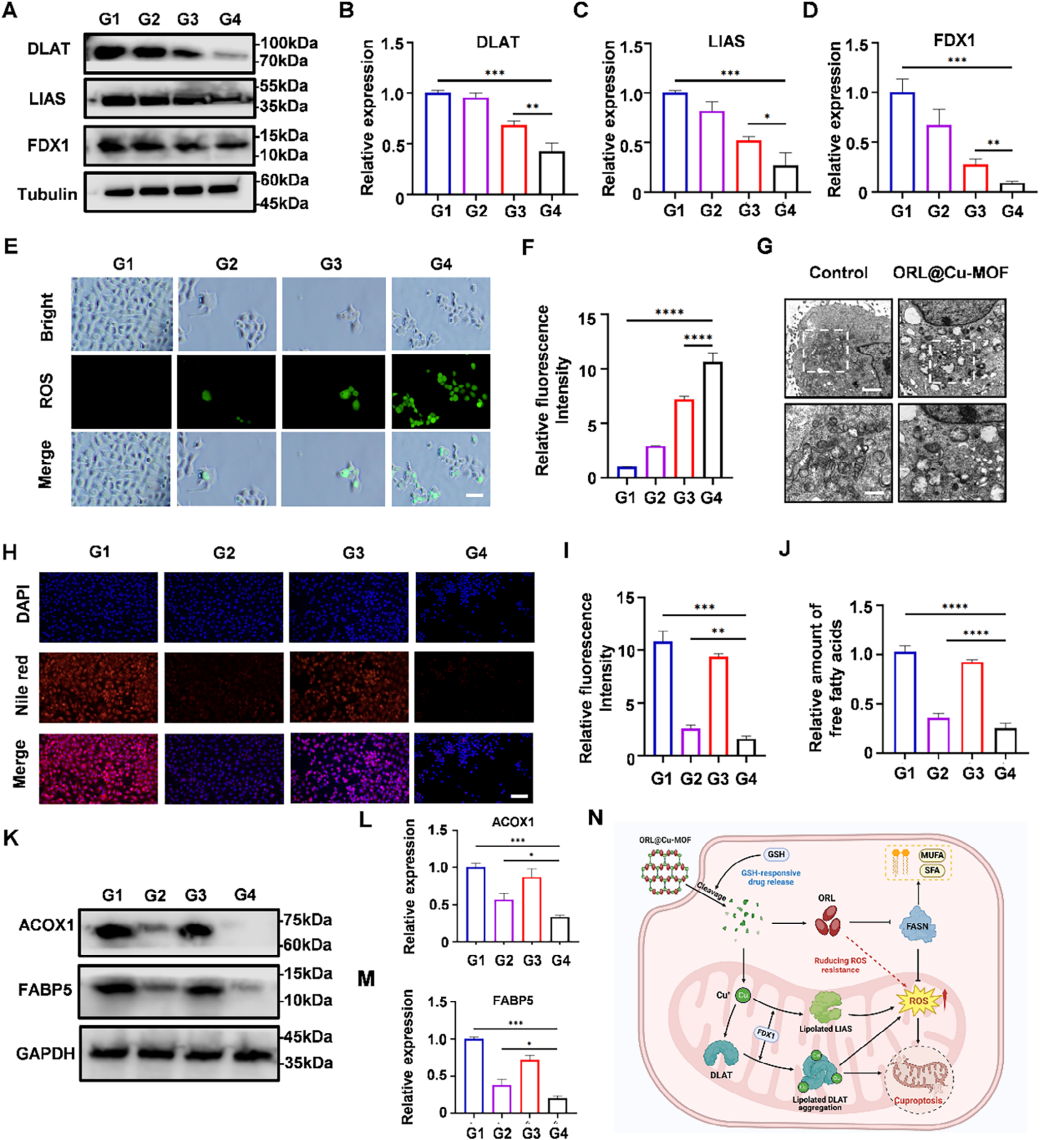
ORL@Cu‐MOF efficiently induces cuproptosis and suppresses fatty acid metabolism. A) Expression levels of DLAT, LIAS, and FDX1 were determined by western blot analysis, with quantification of B) DLAT, C) LIAS, and D) FDX1 protein levels. E) Representative CLSM images and F) semi‐quantification of ROS generation in cells following various treatments; Scale bar, 100 µm. G) Representative bio‐transmission electron microscopy (Bio‐TEM) images of Cal‐27 cells before and after treatment with ORL@Cu‐MOF; Scale bar, upper 2 µm and below 200 nm. H) Representative CLSM images and I) semi‐quantification of lipid droplets in Cal‐27 cells stained with Nile red following different treatments; Scale bar, 50 µm. J) Quantification of free fatty acid content in Cal‐27 cells after various treatments. K) Expression levels of FABP5 and ACOX1 were determined by western blot analysis, with quantification of L) FABP5 and M) ACOX1 protein levels. N) Schematic illustration of the postulated mechanism of action of ORL@Cu‐MOF. G1: Control; G2: ORL; G3: Cu‐MOF; G4: ORL@Cu‐MOF. Data are represented as mean ± SD, *n* = 4. One‐way ANOVA with Tukey's correction for statistical significance. ^*^ *p* < 0.05, ^**^ *p* < 0.01, ^***^ *p* < 0.001, ^****^ *p* < 0.0001.

Cu‐MOF induces downregulation of key cuproptosis proteins DLAT, LIAS, and FDX1, a Hallmark of Cuproptosis.^[^ ^28^ ^]^ To investigate the expression patterns of these proteins upon drug treatment, we employed Western blot analysis. Both Cu‐MOF and ORL@Cu‐MOF treatment resulted in significant downregulation of protein expression levels, whereas ORL treatment did not significantly alter the expression of DLAT, LIAS, and FDX1 (**Figure** **4**A). Notably, the impact of ORL@Cu‐MOF on protein expression was more pronounced than that of Cu‐MOF (Figure 4B–D). This could be attributed to the modulation of fatty acid metabolism in OSCC cells by ORL, which synergizes with the therapeutic effect of Cu‐MOF. Previous studies have demonstrated that cuproptosis leads to an increase in ROS, a crucial mechanism underlying cell death.^[^ ^12^, ^15^ ^]^ To assess intracellular ROS levels in Cal‐27 cells treated with different drugs, we employed the fluorescent probe dichlorodihydrofluorescein diacetate (DCFH‐DA), which is oxidized by ROS to produce the green fluorescent compound dichlorofluorescein. Our results showed that ORL@Cu‐MOF‐treated cells exhibited the highest ROS levels (green), which were 3.2 fold higher than in ORL‐treated cells and ≈1.3 fold higher than in Cu‐MOF‐treated cells (Figure 4E,F). Given that cuproptosis significantly affects mitochondrial function, and this effect is more pronounced in tumor cells compared to normal cells, we examined mitochondrial morphology in Cal‐27 cells treated with ORL@Cu‐MOF using TEM. TEM images revealed mitochondrial shrinkage, increased membrane density, vacuolar changes, and reduction or loss of mitochondrial cristae (Figure 4G).

Consistent with our previous findings that ORL@Cu‐MOF is more effective in inducing cuproptosis than Cu‐MOF, we hypothesized that this effect is mediated by ORL. To further validate this, we speculated that ORL@Cu‐MOF concurrently induces cuproptosis and suppresses fatty acid metabolism in OSCC cells, resulting in a synergistic antitumor effect. To assess whether ORL@Cu‐MOF enhances ORL's inhibitory effect on fatty acid metabolism, we employed Nile Red staining to visualize lipid droplets (red) in Cal‐27 cells (Figure 4H). The results demonstrated that ORL@Cu‐MOF had ≈1.5 fold greater inhibitory effect on lipid synthesis compared to ORL alone (Figure 4I). Additionally, we measured changes in free fatty acid levels and found that ORL@Cu‐MOF treatment resulted in a 1.2 fold decrease in free fatty acid content compared to the ORL group (Figure 4J). ACOX1 and FABP5 are key enzymes in fatty acid metabolism and are closely associated with FASN function.^[^ ^29^, ^30^ ^]^ To further support our hypothesis, we examined the expression levels of ACOX1 and FABP5 and found that ORL@Cu‐MOF treatment significantly downregulated their expression (Figure 4K). Moreover, the effect of ORL@Cu‐MOF was more pronounced than that of ORL alone, while Cu‐MOF did not significantly alter ACOX1 and FABP5 expression levels (Figure 4L,M). Collectively, these findings suggest that ORL@Cu‐MOF effectively induces cuproptosis and modulates fatty acid metabolism in OSCC cells, with a synergistic effect between the two processes (Figure 4N). ORL@Cu‐MOF can induce cuproptosis and suppress fatty acid metabolism.

### 2.6 Antitumor Efficacy of ORL@Cu‐MOF In Vivo

**Figure 5:**
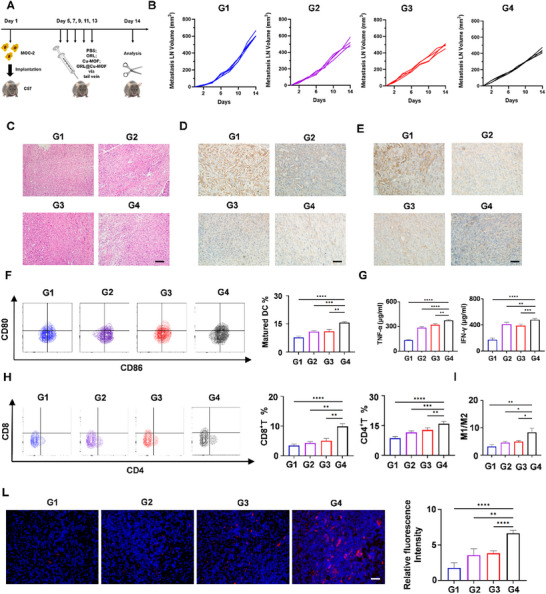
The antitumor activity and remodeling of the tumor immune microenvironment of ORL@Cu‐MOF in vivo. A) Schematic representation of the treatment schedule. B) Growth curves of metastatic LN. C) H&E staining of metastatic LNs from mice receiving different treatments; Scale bar, 100 µm. D) IHC staining of DLAT and E) FASN expression in metastatic LNs during different treatments; Scale bar, 100 µm. F) Representative flow cytometry profiles of matured DC and the percentages of matured DC populations in metastatic LNs following various treatments. G) Evaluation of TNF‐α and IFN‐γ secretion in metastatic LNs using ELISA assay. H) Representative flow cytometry profiles of CD4+T and CD8+T cells, along with their percentages in metastatic LNs after different treatments. I) Quantitative analysis of the ratios of M1/M2 macrophages in the tumors with various treatments. L) Representative immunofluorescence imaging of PD‐1 (red) in metastatic LNs following different treatment regimens; Scale bar, 100 µm. G1: Control; G2: ORL; G3: Cu‐MOF; G4: ORL@Cu‐MOF. Data are represented as mean ± SD, *n* = 4. One‐way ANOVA with Tukey's correction for statistical significance. ^*^ *p* < 0.05, ^**^ *p* < 0.01, ^***^ *p* < 0.001, ^****^ *p* < 0.0001.

To investigate the antitumor effects of ORL@Cu‐MOF in vivo, we established an OSCC LNM model by injecting MOC‐2 cells (a murine‐derived OSCC cell line) into the footpads of mice^[^ ^31^ ^]^ (**Figure** **5**A; Figure S7, Supporting Information). PBS, ORL, Cu‐MOF, and ORL@Cu‐MOF were administered via tail vein injection five consecutive times at a dose of 6 mg kg^−1^ each. Tumor diameters and mouse body weights were recorded every two days from the onset of popliteal/inguinal LN metastasis. We observed that both ORL and Cu‐MOF reduced the burden of LN metastases and slowed the growth rate compared to PBS, while ORL@Cu‐MOF exhibited a more significant therapeutic effect; the volumes of metastatic LNs in mice were 619, 517, 488, and 436 mm^3^ (Figure 5B). Following euthanasia, all metastatic lesions in the popliteal or inguinal regions of the mice were carefully dissected and weighed. The results revealed that the ORL@Cu‐MOF group had the smallest tumor weights, being only 0.50 times that of the PBS‐treated group, 0.59 times that of the ORL group, and 0.66 times that of the Cu‐MOF group (Figure S8A, Supporting Information). Additionally, we investigated changes in body weight across the four groups. Although there were fluctuations in body weight during treatment, no statistically significant differences were observed between the groups (Figure S8B, Supporting Information). Histological analysis of the metastatic LN using H&E staining showed more pronounced nuclear fragmentation and lysis in the ORL@Cu‐MOF‐treated group compared to the other groups, along with fewer mitotic figures and nuclear atypia (Figure 5C). This suggests that ORL@Cu‐MOF may alter the pathological characteristics of the tumor. These results confirm the effective antitumor activity of ORL@Cu‐MOF in vivo.

To investigate whether the antitumor mechanisms of ORL@Cu‐MOF are similar in vitro and in vivo, we stained for the expression of the DLAT in the metastatic LN using IHC. The results demonstrated that ORL@Cu‐MOF effectively reduced DLAT expression in the metastatic LN, being only 68% that of the PBS‐treated group, 88% that of the Cu‐MOF group (Figure 5D and Figure S9, Supporting Information). This indicates that ORL@Cu‐MOF effectively induces cuproptosis in vivo. Furthermore, IHC analysis also revealed that ORL@Cu‐MOF decreased the expression of FASN in the metastatic LNs, with an effect 3.26 times stronger than ORL alone (Figure 5E; Figure S9, Supporting Information). We also quantified free fatty acids levels across treatment groups in vivo. Our data demonstrated that ORL@Cu‐MOF significantly reduced systemic free fatty acids accumulation, with a reduction magnitude superior to that achieved by ORL or Cu‐MOF alone (Figure S10, Supporting Information). Collectively, these results suggest that ORL@Cu‐MOF exerts a synergistic therapeutic effect in vivo by inducing cuproptosis and suppressing fatty acid metabolism.

### 2.7 ORL@Cu‐MOF Remodels the Tumor Microenvironment and Activates Anti‐Tumor Immune Responses

Prior studies have demonstrated that the TME within metastatic LN often exhibits a robust immunosuppressive effect, particularly compromising the process of tumor antigen presentation.^[^ ^8^, ^32^, ^33^ ^]^ To further explore the impact of ORL@Cu‐MOF on the TME, we collected metastatic LN tissues from mice treated with various drugs and employed FCM to investigate the maturation status of DC within the metastatic LN of mice in different treatment groups. The detailed FCM gating approaches and analytical strategies are presented in Figure S11 (Supporting Information), which were adapted from established methodologies in prior studies.^[^ ^15^ ^]^ The results revealed that ORL@Cu‐MOF significantly elevated the proportion of mature DC within metastatic LNs (Figure 5F). Although ORL and Cu‐MOF alone also exhibited a promoting effect on DC maturation, ORL@Cu‐MOF potentiated this effect. This indicates that ORL@Cu‐MOF effectively enhances the efficiency of tumor antigen presentation within metastatic LN. Subsequently, we examined the infiltration of CD4+ T cells and CD8+ T cells and found that the infiltration of CD4+ T cells and CD8+ T cells within metastatic LN of mice treated with ORL@Cu‐MOF was significantly superior to that of other treatment groups (Figure 5H). The detailed FCM gating approaches and analytical strategies are presented in Figure S12 (Supporting Information).

Tumor‐associated macrophages (TAMs) are macrophages infiltrating the tumor microenvironment. Activated macrophages primarily encompass two polarized phenotypes: pro‐inflammatory M1‐type and immunosuppressive M2‐type.^[^ ^34^ ^]^ M1 macrophages (F4/80+CD80+CD206‐) promote T‐cell effector functions through secretion of pro‐inflammatory cytokines/chemokines and antigen presentation. In contrast, M2 macrophages (F4/80+CD80‐CD206+), an immunosuppressive subset, suppress effector T‐cell proliferation.^[^ ^35^, ^36^ ^]^ Consequently, the M1‐to‐M2 polarization shift within the tumor microenvironment (TME) critically facilitates tumor immune evasion and immunosuppression, correlating with poor clinical prognosis.^[^ ^36^, ^37^ ^]^ Our data demonstrated a significantly higher proportion of M1 macrophages in ORL@Cu‐MOF treated murine tumors compared to other treatment groups (Figure S13A, Supporting Information). Conversely, ORL@Cu‐MOF administration markedly reduced M2 macrophage infiltration relative to controls. These findings collectively indicate that ORL@Cu‐MOF reprograms TAMs by inducing M2‐to‐M1 repolarization (Figure 5I; Figure S13B, Supporting Information). In addition, ELISA was used to investigate the levels of TNF‐α and IFN‐γ in metastatic LN treated with different drugs. ORL@Cu‐MOF significantly increased the levels of these anti‐tumor immune cytokines (Figure 5G), which may represent a stronger anti‐tumor immune effect. These findings suggest that ORL@Cu‐MOF ameliorates the immunosuppressive TME within metastatic LN and activates anti‐tumor immune responses. Furthermore, the induction of cuproptosis and inhibition of lipid metabolism synergistically contribute to these improvements. Interestingly, immunofluorescence analysis revealed that ORL@Cu‐MOF elevated the expression level of PD‐1 (red) within metastatic LNs, with a 3.75 fold increase in PD‐1 expression compared to the PBS group (Figure 5L). This provides a theoretical basis for the combination therapy of ORL@Cu‐MOF with αPD‐1.

### 2.8 The Combination of ORL@Cu‐MOF and αPD‐1 can Enhance Antitumor Effects

**Figure 6:**
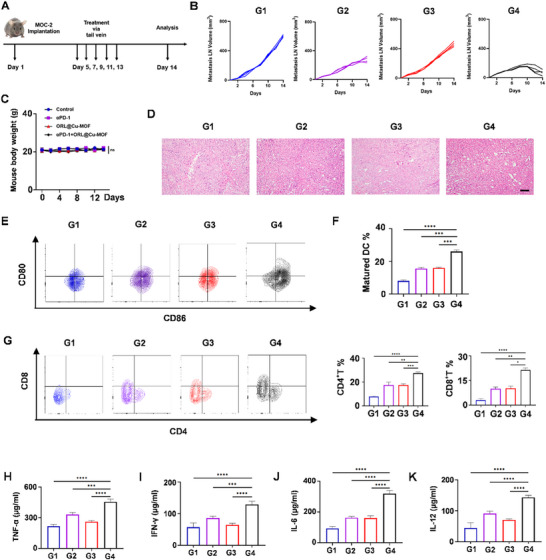
In vivo immune activation and exertion of potent antitumor efficacy with combined ORL@Cu‐MOF and αPD‐1. A)Schematic representation of the treatment schedule. B) Growth curves of metastatic LN. C) Changes in body weight of mice subjected to various treatments. D) H&E staining of metastatic LN from mice receiving different treatments; Scale bar, 100 µm. E) Representative FCM profiles of matured DC and F) the percentages of matured DC populations in metastatic LN following various treatments. G) Representative flow cytometry profiles of CD4+T and CD8+T cells, along with their percentages in metastatic LN after different treatments. Evaluation of the secretion of TNF‐a H), IFN‐γ I), IL‐6 J), and IL‐12 K) in metastatic LNs by ELISA assay. G1: Control; G2: αPD‐1; G3: ORL@Cu‐MOF; G4: αPD‐1+ ORL@Cu‐MOF. Data are represented as mean ± SD, *n* = 4. One‐way ANOVA with Tukey's correction for statistical significance. ^*^ *p* < 0.05, ^**^ *p* < 0.01, ^***^ *p* < 0.001, ^****^ *p* < 0.0001.

Immunotherapy has revolutionized the treatment landscape of OSCC, with αPD‐1 emerging as a frontline therapy for locally advanced or unresectable cases.^[^ ^10^, ^38^ ^]^ Numerous clinical trials targeting αPD‐1 in patients with LNM of OSCC are ongoing. However, only ≈30% of OSCC patients respond effectively to αPD‐1 treatment, reflecting intratumoral heterogeneity, specifically the existence of “cold” and “hot” tumors.^[^ ^9^, ^39^, ^40^ ^]^ Enhancing the responsiveness to αPD‐1 has thus become a critical issue in the management of OSCC with LNM. As previously reported, ORL@Cu‐MOF can modulate the TME within metastatic LNs and enhance tumor immune activation. Consequently, we aimed to explore the antitumor efficacy of combining ORL@Cu‐MOF with αPD‐1. To achieve this, a model of popliteal lymph node metastasis was established by injecting MOC‐2 cells into the footpads of mice. Subsequently, PBS, αPD‐1, ORL@Cu‐MOF, and αPD‐1+ORL@Cu‐MOF (ORL@Cu‐MOF: 6 mg kg^−1^, αPD‐1: 6 mg kg^−1^) were administered to the mice via tail vein injection (**Figures** **6**A and S14, Supporting Information). The volumes of metastatic LNs and body weights of the mice were recorded every two days from the day of drug administration.

After treatment with PBS, αPD‐1, ORL@Cu‐MOF, and αPD‐1+ORL@Cu‐MOF, the volumes of metastatic LNs in mice were 602, 277, 441, and 70 mm^3^, respectively (Figure 6B). Post‐mortem, the metastatic LNs were weighed, revealing that those treated with αPD‐1+ORL@Cu‐MOF weighed only 0.25 grams, demonstrating the excellent therapeutic effect of this combination (Figure S15, Supporting Information). Notably, no abrupt changes in body weight were observed during treatment, and there were no statistically significant differences in body weights among the groups (Figure 6C). Histological analysis of the metastatic LNs using H&E staining revealed that αPD‐1+ORL@Cu‐MOF treatment resulted in more nuclear fragmentation and fewer nuclear mitoses and nuclear atypia compared to PBS, αPD‐1, and ORL@Cu‐MOF alone (Figure 6D).

To further investigate the immunomodulatory effects of αPD‐1 combined with ORL@Cu‐MOF, metastatic LNs and draining lymph nodes (TDLN) from the groin region were collected, and immune‐related parameters were analyzed. FCM analysis revealed that αPD‐1+ORL@Cu‐MOF treatment significantly increased the number of mature DC in metastatic LNs (Figure 6E,F). This enhancement suggests a stronger antigen‐presenting capacity. Furthermore, the proportions of CD4+ T and CD8+ T cells in metastatic LNs treated with αPD‐1+ORL@Cu‐MOF were increased by significantly increased compared to those treated with PBS, respectively (Figure 6G).

To support this claim, we also examined the levels of the antitumor immune response factors TNF‐α, IFN‐γ, and IL‐6 within the tumor.^[^ ^41^ ^]^ The αPD‐1+ORL@Cu‐MOF treatment significantly increased the levels of these immune factors (Figure 6H–K). The upregulation of these factors represents a more effective antitumor immune response, and the tumor immune level induced by αPD‐1+ORL@Cu‐MOF is higher than that of αPD‐1 alone. This indicates that ORL@Cu‐MOF can enhance the antitumor effects of αPD‐1, effectively converting “cold tumors” into “hot tumors”.

### 2.9 Biodistribution and Biosafety of ORL@Cu‐MOF

**Figure 7:**
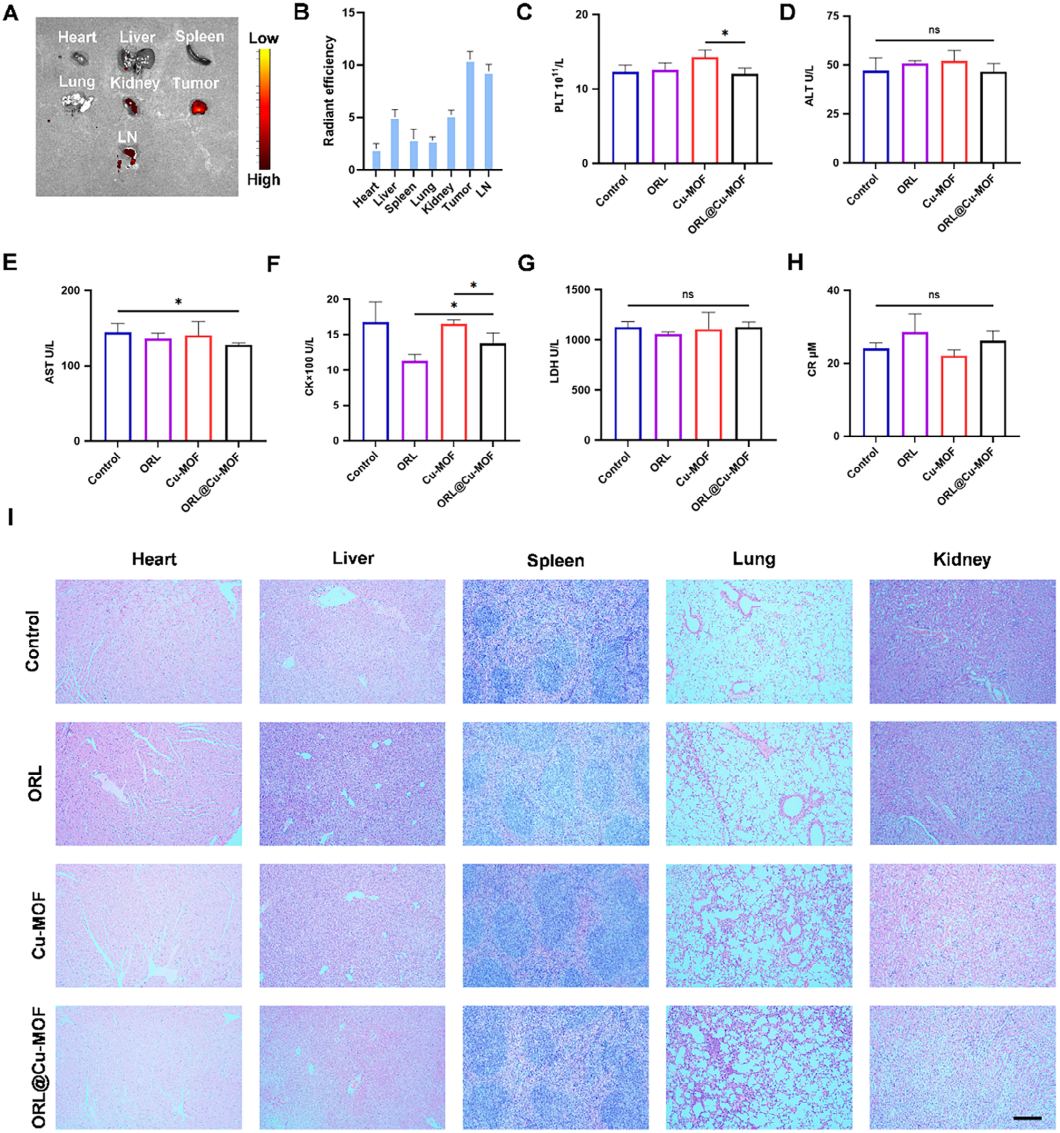
Biological safety of ORL@Cu‐MOF. A) Ex *vivo* imaging and B) MFI of ORL@Cu‐MOF@Cy7.5 in major organs and tumor. C–H) Biochemical analysis of serum from mice receiving various treatments: (C) platelet count (PLT); (D) alanine aminotransferase (ALT); (E) aspartate aminotransferase (AST); (F) creatine kinase (CK); (G) lactate dehydrogenase (LDH); and (H) creatinine (CR). I) Evaluation of major organs through H&E staining; Scale bar, 100 µm. Data are represented as mean ± SD, *n* = 4. One‐way ANOVA with Tukey's correction for statistical significance. ^*^ *p* < 0.05, ns, not significant.

To assess the biosafety of ORL@Cu‐MOF, mice were injected with PBS, ORL, Cu‐MOF, and ORL@Cu‐MOF (6 mg kg^−1^) via the tail vein, with administrations occurring every two days. Mice were sacrificed after five administrations. 24 h post intravenous administration of Cy7.5‐labeled ORL@Cu‐MOF (ORL@Cu‐MOF@Cy7.5) via the tail vein, mice were euthanized to investigate the ex vivo biodistribution of the nanocomposite. Results revealed the most intense fluorescence signals in tumor and LN, surpassing heart, liver, spleen, lung, and kidney accumulation (**Figure** **7**A), indicative of robust tumor‐targeting capability (Figure 7B). Collectively, these data demonstrate that ORL@Cu‐MOF achieves rapid tumor‐targeting accumulation, which critically underpins its therapeutic utility. Biochemical analysis of blood samples collected from these mice revealed no significant differences in alanine aminotransferase, aspartate aminotransferase, creatine kinase, total bilirubin, direct bilirubin, urea, and serum creatinine levels between mice treated with ORL@Cu‐MOF and those treated with PBS (Figure 7C–H; Figures S16–S18, Supporting Information). Subsequently, hearts, livers, spleens, lungs, and kidneys were collected from mice treated with different drugs and stained with H&E. Notably, histological analysis of major organs and tissues from mice treated with ORL@Cu‐MOF revealed no significant morphological changes (Figure 7I), indicating that ORL@Cu‐MOF does not cause significant toxic reactions. In summary, ORL@Cu‐MOF exhibits low toxicity and sufficient biosafety for in vivo applications.

## 3 Conclusion 

Cuproptosis, a newly discovered immunogenic form of cell death, relies on the transport of copper ions to elicit oxidative stress, ultimately triggering cellular demise. Tumor cells, particularly those exhibiting metastatic properties, fortify their resilience against oxidative stress through lipid metabolic reprogramming. Notably, alterations in fatty acid metabolism play a crucial role in this process, significantly contributing to the challenges in treating metastatic cancers and the poor prognosis for patients.

In our study, we have developed a copper‐based MOF loaded with ORL, designated as ORL@Cu‐MOF. This nanomaterial exhibits remarkable stability and a GSH‐responsive release capability, enabling it to effectively induce cuproptosis and suppress fatty acid metabolism both in vitro and in vivo. ORL@Cu‐MOF downregulation of cuproptosis‐related proteins, such as DLAT, LIAS, and FDX1, along with its ability to attenuate fatty acid metabolism and reshape lipid metabolic reprogramming in OSCC cells through the inhibition of FASN, underscores its therapeutic potential. ORL@Cu‐MOF demonstrates the ability to remodel the TME, reversing its immunosuppressive nature and converting “cold tumors” into immunogenic “hot tumors”. Additionally, this nanodrug enhances the expression of PD‐1 within the LNM tissues, offering a novel approach to enhancing immunotherapy's efficacy. Combination of ORL@Cu‐MOF with αPD‐1 addresses the challenges associated with the low response rates observed in αPD‐1 therapy, significantly potentiating its therapeutic effects. This synergistic approach not only provides a promising new treatment strategy for OSCC but also holds great promise in the management of other cancers and metastatic diseases.

In conclusion, we have established a paradigm‐shifting nanomedicine approach that combines the induction of cuproptosis with the suppression of fatty acid metabolism to tackle the challenging issue of LNM in OSCC. The integration of ORL@Cu‐MOF with αPD‐1 represents a significant step forward in enhancing the effectiveness of immunotherapy and offers hope for improved outcomes in the treatment of various cancers.

## Conflict of Interest

The authors declare no conflict of interest.

## Author Contributions

Z.Z.L. and Y.L. contributed equally to this work. Z.‐Z.L. and Y.L. performed conceptualization, visualization, methodology, investigation, wrote, reviewed, and edited the final manuscript. L.‐M.C. and K.Z. performed methodology, investigation, and wrote the original draft. G.‐R.W. and Y.X. performed methodology, formal analysis, and visualization. J.W. and Y.‐F.Y. performed the methodology and wrote the original draft. B.L. and Q.W. performed, wrote, reviewed, and edited the final manuscript, project administration, and funded acquisition. Z.S. and L.‐L.B. performed conceptualization, methodology, project administration, funded acquisition, wrote, reviewed, and edited the final manuscript. All authors reviewed and approved the final manuscript.

## Ethics Statement

All animal experimental procedures were performed in accordance with the guidelines provided by the National Institutes of Health Guide for the Care and Use of Laboratory Animals. All animal experiments reported herein were performed under the guidelines evaluated and approved by the Committee of Animal Experimentation and the Ethics Committee of the School of Stomatology, Wuhan University (Approval number: 50792403002). The mice were housed in accordance with animal welfare regulations, under specific‐pathogen‐free conditions at 25 °C, 50% humidity, and a 12 h light/dark cycle.

## Supporting information



Supporting Information

## Data Availability

The data that support the findings of this study are available from the corresponding author upon reasonable request.

## References

[1] V. Bouvard , S. T. Nethan , D. Singh , S. Warnakulasuriya , R. Mehrotra , A. K. Chaturvedi , T. H. H. Chen , O. A. Ayo‐Yusuf , P. C. Gupta , A. R. Kerr , W. M. Tilakaratne , D. Anantharaman , D. Conway , A. Gillenwater , N. W. Johnson , L. P. Kowalski , M. E. Leon , O. Mandrik , T. Nagao , V. M. Prasad , K. Ramadas , F. Roitberg , P. Saintigny , R. Sankaranarayanan , A. R. Santos‐Silva , D. N. Sinha , P. Vatanasapt , R. B. Zain , B. Lauby‐Secretan , N. Engl. J. Med. 2022, 21, 387, 10.1056/NEJMsr2210097 36378601

[2] L. Q. M. Chow , N. Engl. J. Med. 2020, 382, 62. 10.1056/NEJMc2001370 32402180

[3] Z. Z. Li , Z. M. Cai , W. T. Zhu , N. N. Zhong , L. M. Cao , G. R. Wang , Y. Xiao , Z. Q. Zhu , X. H. Liu , K. Wu , R. X. He , X. Z. Zhao , B. Liu , B. Cai , L. L. Bu , J. Nanobiotechnol. 2024, 22, 586. 10.1186/s12951-024-02846-1 PMC11437730 39342329

[4] P. M. Speight , P. M. Farthing , Br. Dent. J. 2018, 225, 9. 10.1038/sj.bdj.2018.925 30412570

[5] Z. Z. Li , K. Zhou , Q. J. Wu , B. Liu , L. L. Bu , Crit Rev Oncol Hemat 2024, 204 , 104536. 10.1016/j.critrevonc.2024.104536 39426554

[6] Z. M. Cai , Z. Z. Li , N. N. Zhong , L. M. Cao , Y. Xiao , J. Q. Li , F. Y. Huo , B. Liu , C. Xu , Y. Zhao , L. Rao , L. L. Bu , J Nanobiotechnology 2024, 22 , 135. 38553735 10.1186/s12951-024-02408-5 PMC10979629

[7] H. S. Deng , J. Zhou , H. L. Chen , X. Y. Cai , R. Zhong , F. Li , B. Cheng , C. C. Li , Q. Z. Jia , C. C. Zhou , R. H. Petersen , G. Rocco , A. Brunelli , C. S. H. Ng , T. A. D'Amico , C. X. Su , J. X. He , W. H. Liang , B. Zhu , A. T. S. Collaborative , International Journal of Surgery 2024, 110, 1. 10.1097/JS9.0000000000000774 PMC10793742 37755384

[8] M. K. Rahim , T. L. H. Okholm , K. B. Jones , E. E. McCarthy , C. C. Liu , J. L. Yee , S. J. Tamaki , D. M. Marquez , I. Tenvooren , K. Wai , A. Cheung , B. R. Davidson , V. Johri , B. Samad , W. E. O'Gorman , M. F. Krummel , A. van Zante , A. J. Combes , M. Angelo , L. Fong , A. P. Algazi , P. Ha , M. H. Spitzer , Cell 2023, 186 , 6. 10.1016/j.cell.2023.02.021 PMC10348701 36931243

[9] K. Balachander , A. Paramasivam , Oral Oncol 2022, 132 , 105997. 35772187 10.1016/j.oraloncology.2022.105997

[10] Y. Xiao , Z. Z. Li , N. N. Zhong , L. M. Cao , B. Liu , L. L. Bu , Transl Oncol 2023, 38 , 101794. 37820473 10.1016/j.tranon.2023.101794 PMC10582482

[11] R. Xie , Y. F. Wang , F. Tong , W. Q. Yang , T. Lei , Y. F. Du , X. R. Wang , Z. X. Yang , T. Gong , M. Shevtsov , H. L. Gao , Small 2023, 19 , 37. 10.1002/smll.202300570 37222118

[12] P. Tsvetkov , S. Coy , B. Petrova , M. Dreishpoon , A. Verma , M. Abdusamad , J. Rossen , L. Joesch‐Cohen , R. Humeidi , R. D. Spangler , J. K. Eaton , E. Frenkel , M. Kocak , S. M. Corsello , S. Lutsenko , N. Kanarek , S. Santagata , T. R. Golub , Science 2022, 375 , 6586. 10.1126/science.abf0529 PMC9273333 35298263

[13] Y. Li , J. Liu , Y. Chen , R. R. Weichselbaum , W. Lin , Adv. Sci. (Weinh) 2024, 11, 23. 10.1002/advs.202310309 PMC11187894 38477411

[14] V. Oliveri , Coordin Chem Rev 2020, 422 , 56.

[15] B. D. Guo , F. Y. Yang , L. P. Zhang , Q. X. Zhao , W. K. Wang , L. Yin , D. Chen , M. S. Wang , S. J. Han , H. H. Xiao , N. Z. Xing , Adv. Mater. 2023, 35 , 22. 10.1002/adma.202212267 36916030

[16] Y. Z. Xu , S. Y. Liu , L. L. Zeng , H. S. Ma , Y. F. Zhang , H. H. Yang , Y. C. Liu , S. Fang , J. Zhao , Y. S. Xu , C. J. Ashby , Y. L. He , Z. Dai , Y. H. Pan , Adv. Mater. 2023, 35, 1.

[17] M. Luo , J. Yan , X. Hu , H. Li , H. Li , Q. Liu , Y. Chen , Z. Zou , Apoptosis 2023, 28, 1. 36399287 10.1007/s10495-022-01795-0

[18] Y. Chen , Y. Dai , K. Song , Y. Huang , L. Zhang , C. Zhang , Q. Yan , H. Gao , Cell Death Dis. 2021, 12 , 6. 10.1038/s41419-021-03850-1 PMC8175732 34083513

[19] Y. Chen , Y. Feng , Y. Lin , X. Zhou , L. Wang , Y. Zhou , K. Lin , L. Cai , Br. J. Cancer 2024, 130, 755. 38228715 10.1038/s41416-024-02574-1 PMC10912431

[20] J. R. Gale , K. Hartnett‐Scott , M. M. Ross , P. A. Rosenberg , E. Aizenman , J. Neurochem. 2023, 167 , 2. 10.1111/jnc.15961 PMC10591933 37702109

[21] L. Luo , A. Li , S. Fu , W. Du , L. N. He , X. Zhang , Y. Wang , Y. Zhou , Y. Yunpeng , Z. Li , S. Hong , Immunol Res 2023, 71 , 2. 10.1007/s12026-022-09335-3 36434349

[22] Y. N. Dai , D. L. Leng , Z. Guo , J. Q. Wang , Y. H. Gu , Y. J. Peng , L. P. Zhu , Q. Zhao , Chem. Eng. J. 2024, 479, 9.

[23] K. R. Chen , A. W. Zhou , X. Y. Zhou , Y. H. Liu , Y. R. Xu , X. H. Ning , Nano Lett. 2023, 23, 7. 10.1021/acs.nanolett.3c00434 36951267

[24] S. R. Li , L. L. Bu , L. Cai , Signal Transduct Target Ther 2022, 1, 7. 10.1038/s41392-022-01014-x PMC9106713 35562341

[25] P. Zhang , J. H. Zhao , L. X. Yuan , L. L. Ju , H. X. Wang , F. Wang , L. Chen , W. H. Cai , Sci. Rep. 2023, 13, 1. 37828099 10.1038/s41598-023-43835-y PMC10570290

[26] Q. Xue , R. Kang , D. J. Klionsky , D. Tang , J. Liu , X. Chen , Autophagy 2023, 19 , 8. 10.1080/15548627.2023.2200554 PMC10351475 37055935

[27] K. R. Chen , A. W. Zhou , X. Y. Zhou , J. L. He , Y. R. Xu , X. H. Ning , Sci. Adv. 2024, 10, 15. 10.1126/sciadv.adk3201 PMC11006215 38598629

[28] B. Liu , J. N. Liu , G. J. Chen , C. Xu , L. L. Bu , Front Cell Dev Biol 2023, 11, 1307501. 38077997 10.3389/fcell.2023.1307501 PMC10704899

[29] C. W. Fhu , A. Ali , Molecules 2020, 25 , 17. 32872164 10.3390/molecules25173935 PMC7504791

[30] C. Zhang , Y. Liao , P. Liu , Q. Du , Y. Liang , S. Ooi , S. Qin , S. He , S. Yao , W. Wang , Theranostics 2020, 10, 15. 10.7150/thno.44868 PMC7295046 32550890

[31] M. Agostini , L. Y. Almeida , D. C. Bastos , R. M. Ortega , F. S. Moreira , F. Seguin , K. G. Zecchin , H. F. Raposo , H. C. F. Oliveira , N. D. Amoêdo , T. Salo , R. D. Coletta , E. Graner , Mol. Cancer Ther. 2014, 13, 3. 24362464 10.1158/1535-7163.MCT-12-1136

[32] K. M. van Pul , R. Vuylsteke , R. van de Ven , E. A. Te Velde , E. J. T. Rutgers , P. M. van den Tol , H. Stockmann , T. D. de Gruijl , J Immunother Cancer 2019, 7, 1. 31118093 10.1186/s40425-019-0605-1 PMC6530094

[33] N. E. Reticker‐Flynn , W. R. Zhang , J. A. Belk , P. A. Basto , N. K. Escalante , G. O. W. Pilarowski , A. Bejnood , M. M. Martins , J. A. Kenkel , I. L. Linde , S. Bagchi , R. B. Yuan , S. Chang , M. H. Spitzer , Y. Carmi , J. H. Cheng , L. L. Tolentino , O. Choi , N. Wu , C. S. Kong , A. J. Gentles , J. B. Sunwoo , A. T. Satpathy , S. K. Plevritis , E. G. Engleman , Cell 2022, 185, 11. 10.1016/j.cell.2022.04.019 PMC9149144 35525247

[34] N. Kumari , S. H. Choi , J Exp Clin Cancer Res 2022, 41, 1. 35183252 10.1186/s13046-022-02272-x PMC8857848

[35] Y. Wang , G. Li , J. Su , Y. Liu , X. Zhang , G. Zhang , Z. Wu , J. Li , X. Wang , Y. Zhang , M. Bai , Y. Yao , R. Wang , K. Shao , Small 2025, 21, 6. 10.1002/smll.202406839 39797442

[36] X. Zhang , G. Li , J. Yin , W. Pan , Y. Li , N. Li , B. Tang , Nano Lett. 2024, 24, 29. 10.1021/acs.nanolett.4c02657 39007505

[37] L. Niu , Q. Wang , F. Feng , W. Yang , Z. Xie , G. Zheng , W. Zhou , L. Duan , K. Du , Y. Li , Y. Tian , J. Chen , Q. Xie , A. Fan , H. Dan , J. Liu , D. Fan , L. Hong , J. Zhang , J. Zheng , Biochim Biophys Acta Mol Basis Dis 2024, 1870, 2. 10.1016/j.bbadis.2023.166917 37820821

[38] Z. Z. Li , N. N. Zhong , L. M. Cao , Z. M. Cai , Y. Xiao , G. R. Wang , B. Liu , C. Xu , L. L. Bu , Small 2024, 20 , 19. 10.1002/smll.202308731 38327169

[39] Z. Y. Xu , Z. Z. Li , L. M. Cao , N. N. Zhong , X. H. Liu , G. R. Wang , Y. Xiao , B. Liu , L. L. Bu , Cancer Lett 2024, 588, 216740. 38423247 10.1016/j.canlet.2024.216740

[40] K. Zhou , Z. Z. Li , Z. M. Cai , N. N. Zhong , L. M. Cao , F. Y. Huo , B. Liu , Q. J. Wu , L. L. Bu , Pharmacol Res 2023, 198, 1. 10.1016/j.phrs.2023.106989 37979662

[41] J. L. Liang , X. K. Jin , S. M. Zhang , Q. X. Huang , P. Ji , X. C. Deng , S. X. Cheng , W. H. Chen , X. Z. Zhang , Sci Bull (Beijing) 2023, 68, 6. 10.1016/j.scib.2023.02.027 36914548

