# Supplementary material for: ORL@Cu‐MOF Boost Cuproptosis and Suppress Fatty Acid Metabolism for Cancer Lymph Node Metastasis Synergistic Therapy
Source: Adv Sci (Weinh). 2025 Jun 23;12(35):e02154. doi: 10.1002/advs.202502154 (PMC12463119; doi:10.1002/advs.202502154)
Supplement: Supplementary file 1 — Supporting Information [file ADVS-12-e02154-s001.docx]

**ORL@Cu-MOF Boost Cuproptosis and Suppress Fatty Acid Metabolism for Cancer Lymph Node Metastasis Synergistic Therapy**

Zi-Zhan Li^a,b, #^, Yi Liu^c,d, #^, Kan Zhou^a^, Lei-Ming Cao^a^, Guang-Rui Wang^a^, Jinmei Wu^c^, Yi-Fu Yu^a^, Yao Xiao^a^, Bing Liu^a,b,^ *, Qiuji Wu^e,^ *, Zhiyong Song^c,^ *, Lin-Lin Bu^a,b,^ *

^a^ State Key Laboratory of Oral & Maxillofacial Reconstruction and Regeneration, Key Laboratory of Oral Biomedicine Ministry of Education, Hubei Key Laboratory of Stomatology, School & Hospital of Stomatology, Wuhan University, Wuhan, Hubei, 430079, China

^b^ Department of Oral & Maxillofacial - Head Neck Oncology, School & Hospital of Stomatology, Wuhan University, Wuhan, Hubei, 430079, China

^c^ National Key Laboratory of Agricultural Microbiology, College of Chemistry, Huazhong Agricultural University, Wuhan, 430070, China

^d^ Key Laboratory of Combinatorial Biosynthesis and Drug Discovery, School of Pharmaceutical Sciences, Wuhan University, Wuhan, 430071, China.

^e^ Department of Radiation and Medical Oncology, Hubei Key Laboratory of Tumor Biological Behaviors, Hubei Cancer Clinical Study Center, Zhongnan Hospital of Wuhan University, Wuhan, China

^#^ Contribute equally

^*^ Corresponding authors:

Bing Liu, MD, Email: liubing9909@whu.edu.cn

Qiuji Wu, MD, Email: wuqiuji@znhospital.cn

Zhiyong Song, PhD, Email: songzhiyong@mail.hzau.edu.cn

Lin-Lin Bu, MD, PhD, Email: lin-lin.bu@whu.edu.cn


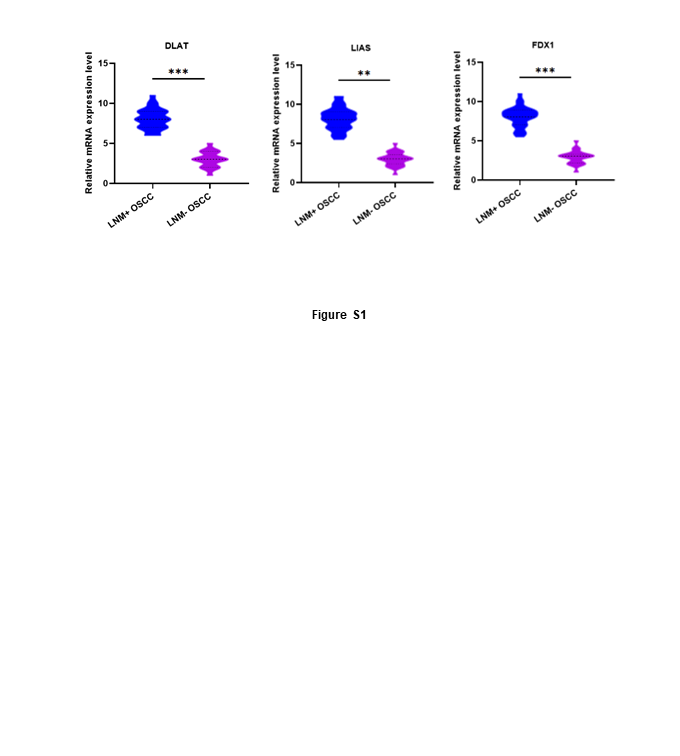


Figure S1. The relative expression levels of DLAT, LIAS, and FDX1 mRNA in LNM+ OSCC patients and LNM- OSCC patients. ***p* < 0.01, ****p* < 0.001.


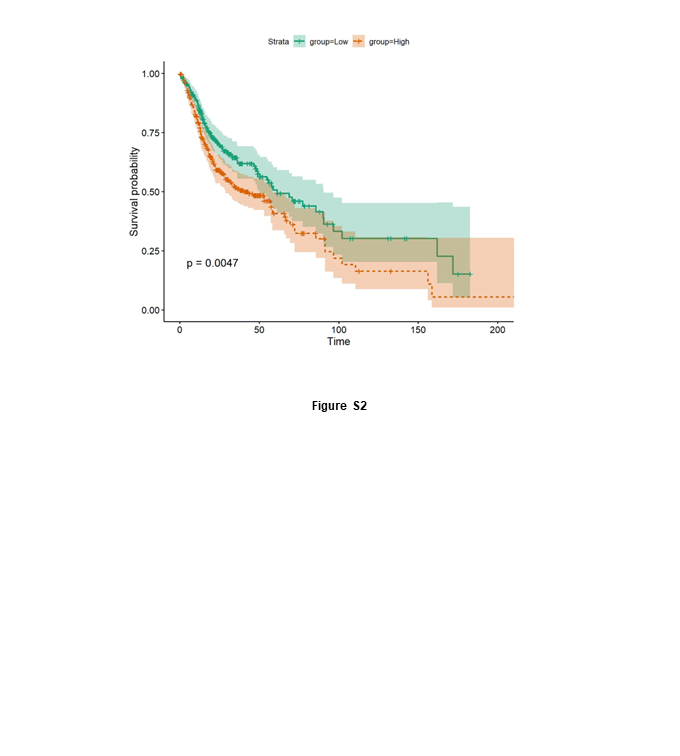


Figure S2. The relationship between the expression level of DLAT and the survival of OSCC patients.


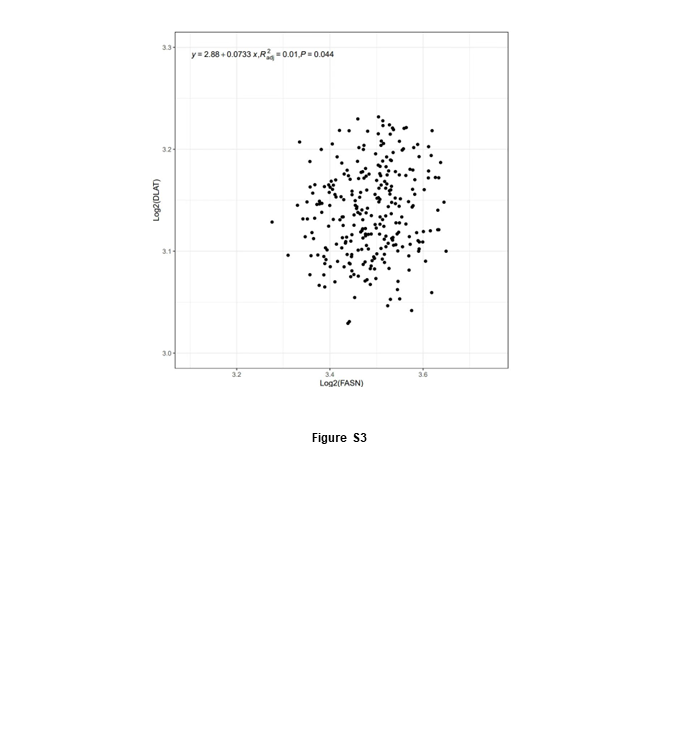


Figure S3. The correlation between DLAT and FASN expression levels in OSCC patients.


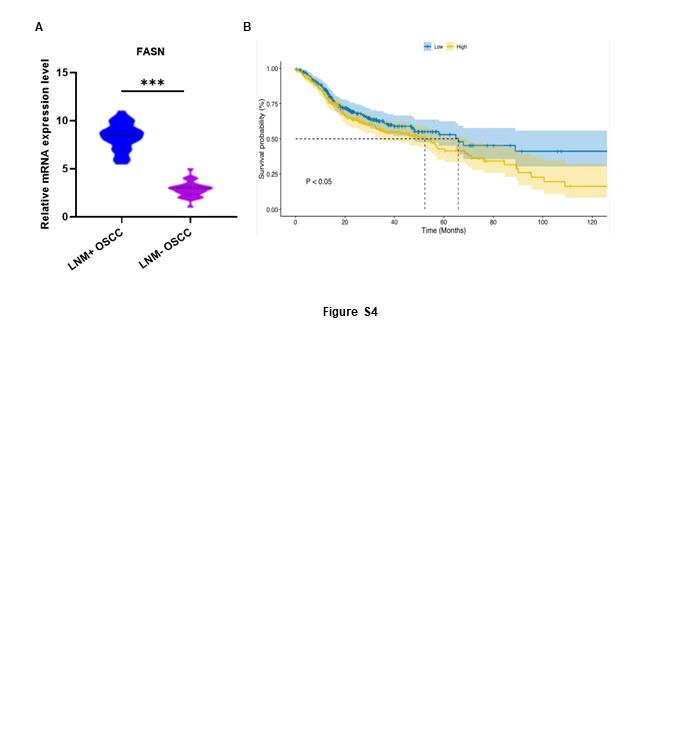


Figure S4. (A) The relative expression levels of FASN mRNA in LNM+ OSCC patients and LNM- OSCC patients. (B) The relationship between the expression level of FASN and the survival of OSCC patients. ****p* < 0.001.


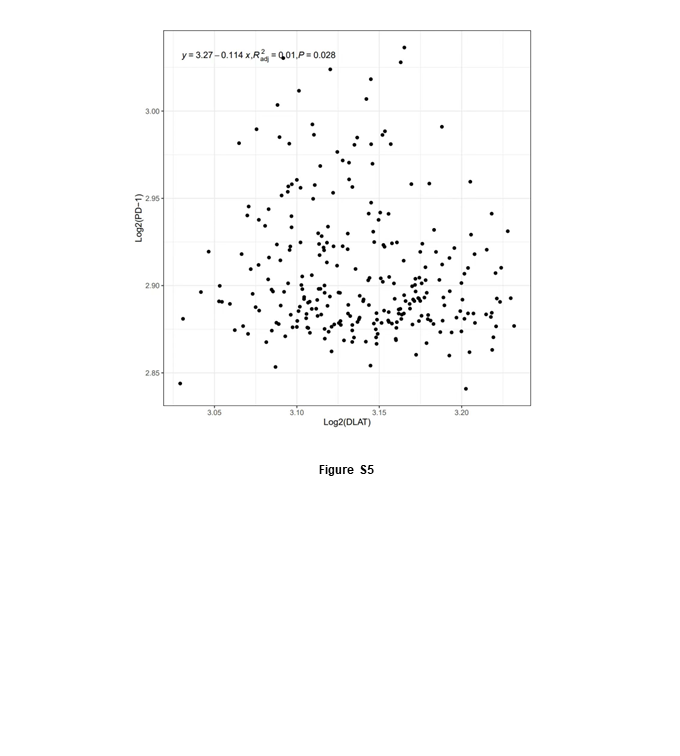


Figure S5. The correlation between DLAT and PD-1 expression levels in OSCC patients.


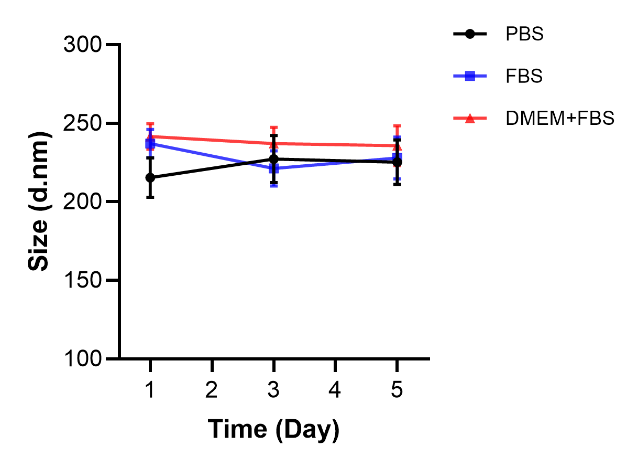


Figure S6. Stability of different ORL@Cu-MOF NPs in PBS, FBS, and DMEM+FBS.


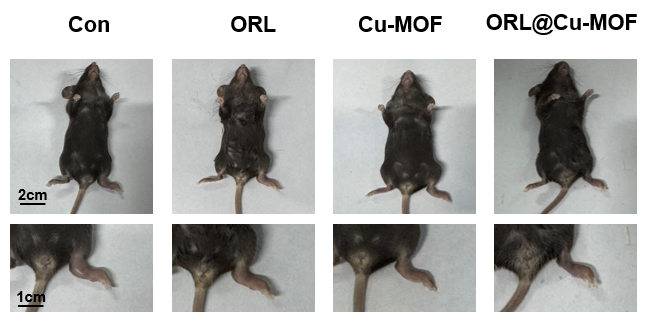


Figure S7. Animal model of popliteal lymph node metastasis induced by injection of MOC-2 cells into foot pads via different treatments.


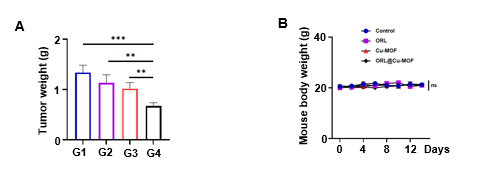


Figure S8. (A) In *vivo* metastatic LN weight, along with (B) changes in body weight of mice subjected to various treatments. G1: Control; G2: ORL; G3: Cu-MOF; G4: ORL@Cu-MOF. ***p* < 0.01, ****p* < 0.001.


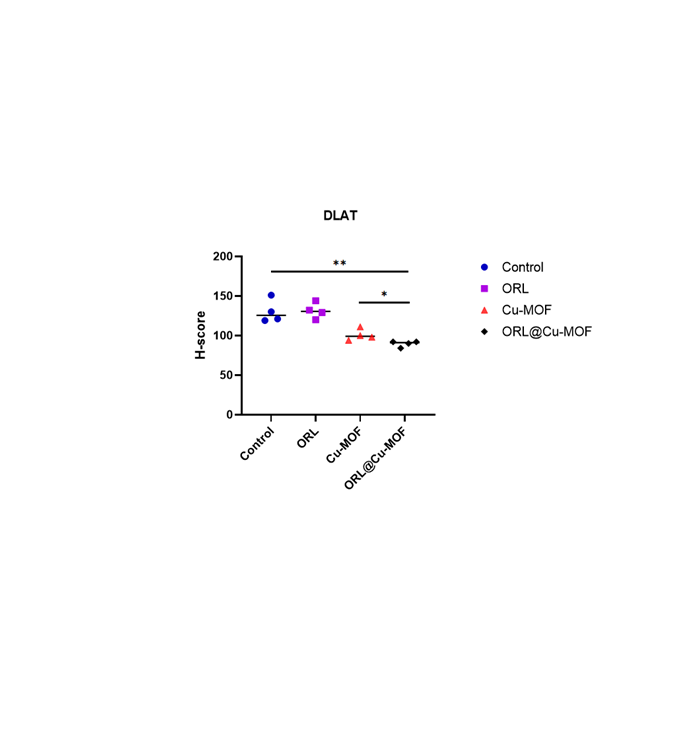

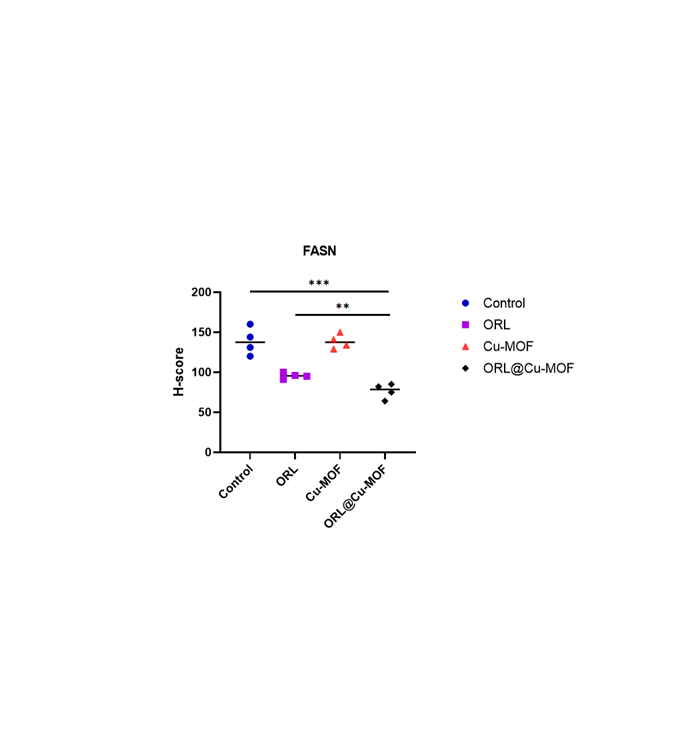


Figure S9. Immunohistochemical H-score of DLAT and FASN in metastatic LN after different treatments.


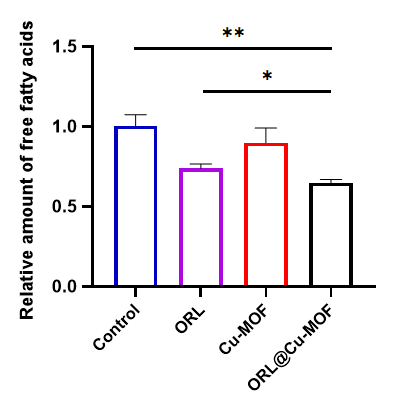


Figure S10. The content of free fatty acid in *vivo* after various treatments.


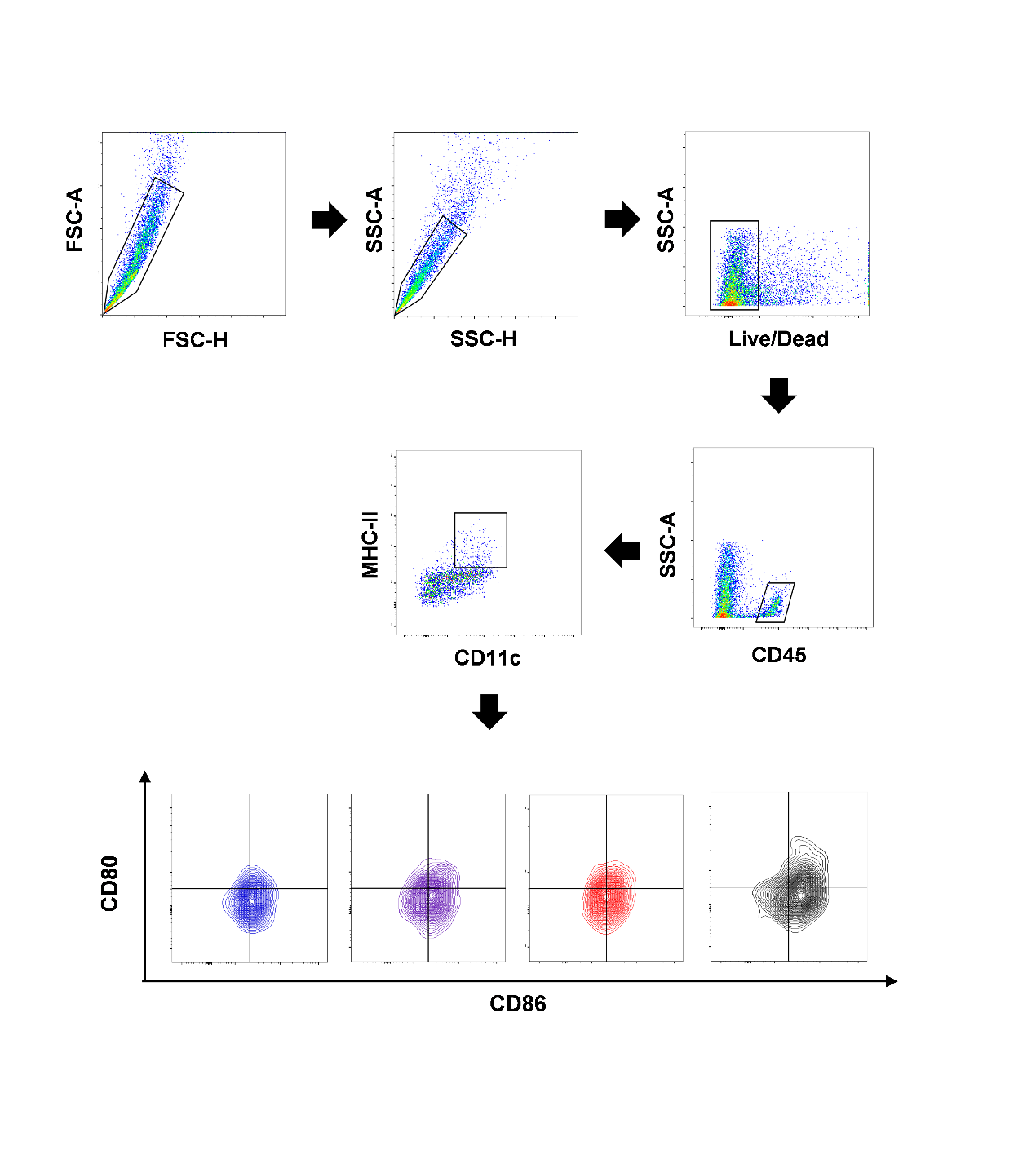


Figure S11. The gating strategy of mature DCs in tumors after various treatments, wherein matured DCs were denoted as CD80+CD86+ cells.


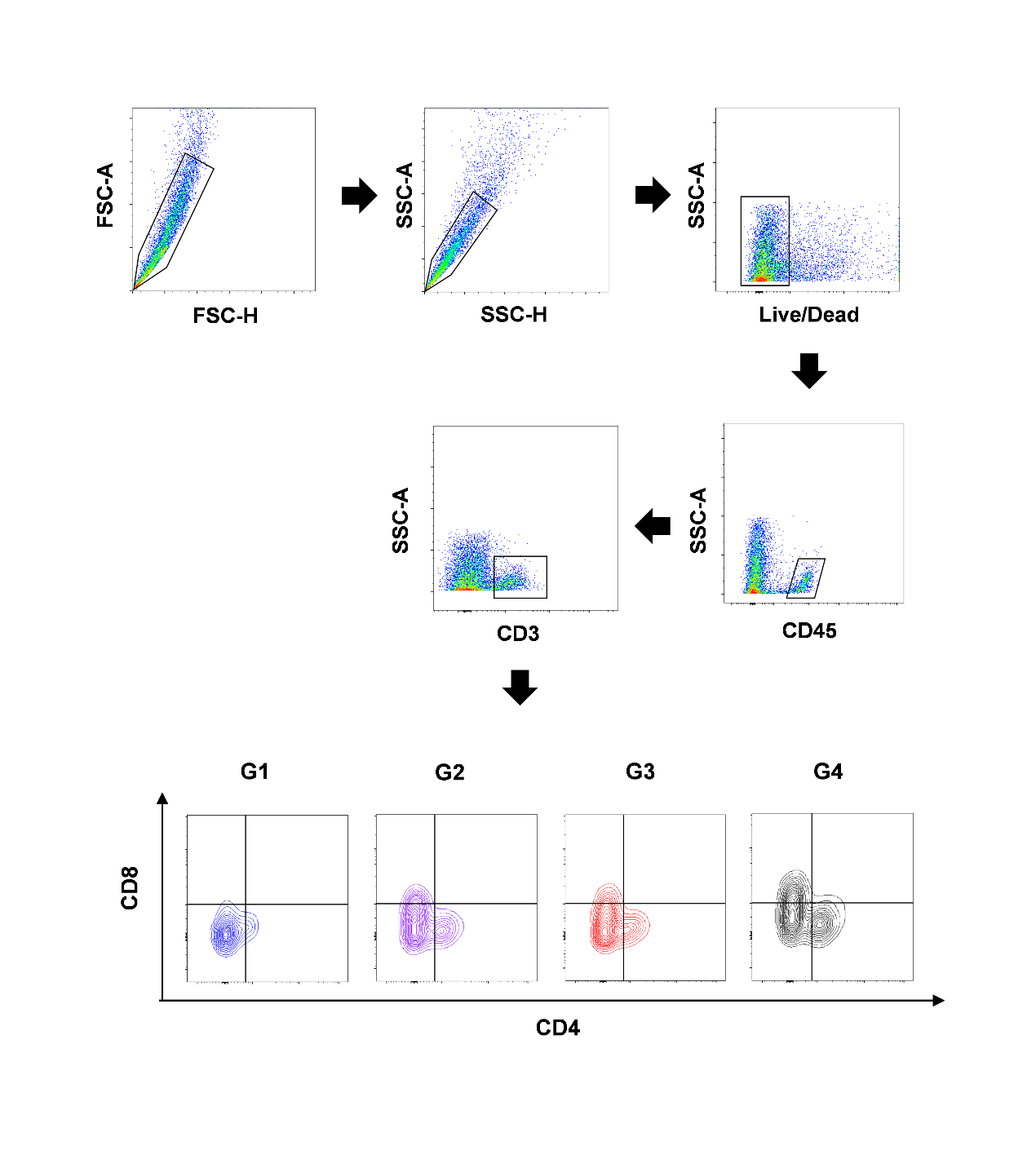


Figure S12. The gating strategy of CD3+CD8+ T and CD3+CD4+ T cells in tumors after various treatments, which was denoted as the percentage of CD4+ T and CD8+ T cells in the population of CD3+ T cells.


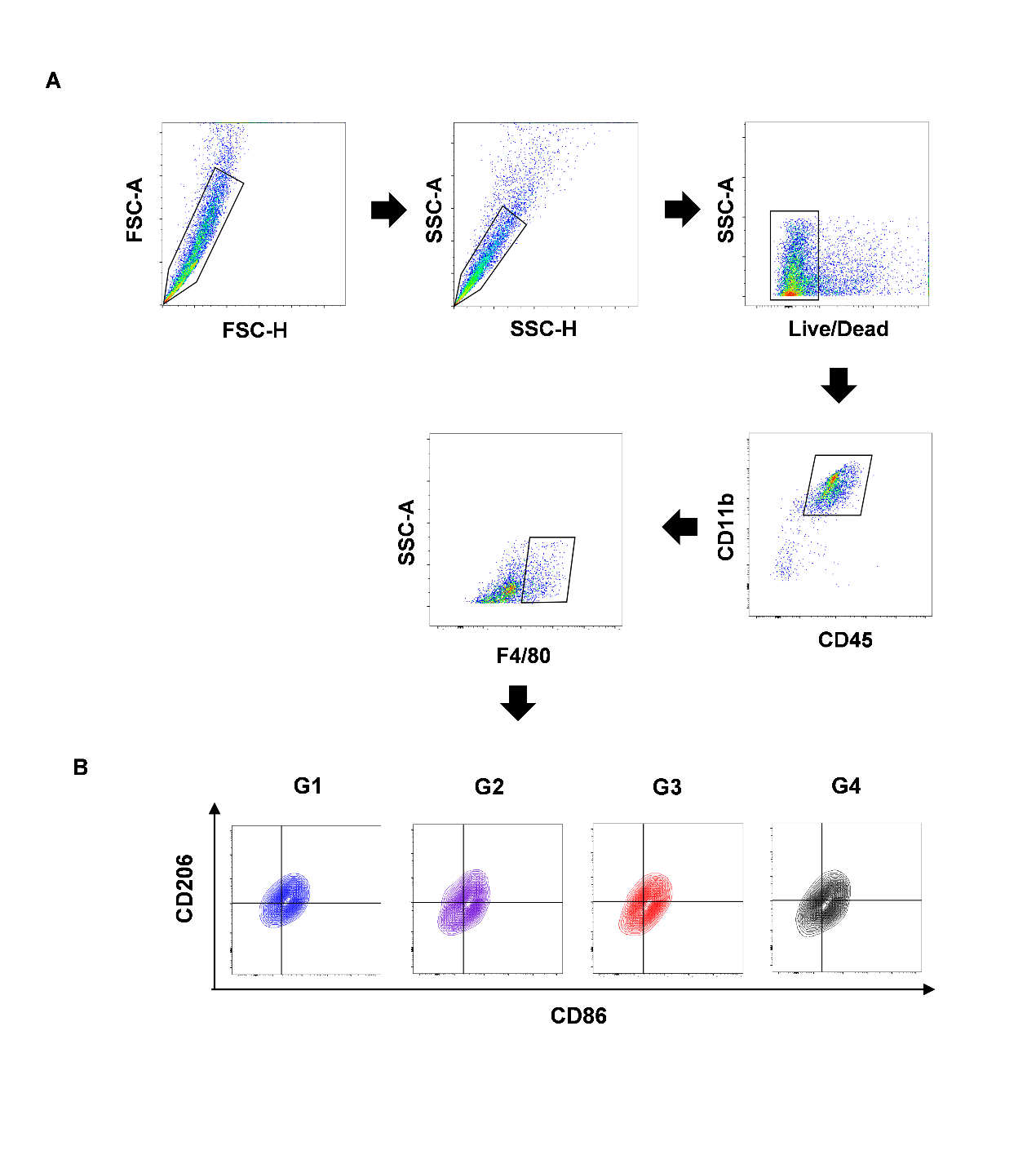


Figure S13. (A) The gating strategies of M1 and M2-phenotype macrophages in tumors after various treatments. Macrophages were denoted as F4/80 positive cells, wherein M1phenotype macrophages were F4/80+CD80+CD206- cells and M2-phenotype macrophages were F4/80+CD80-CD206+ cells. (B) Representative FCM profiles of M1 and M2-phenotype macrophages in tumors after various treatments.


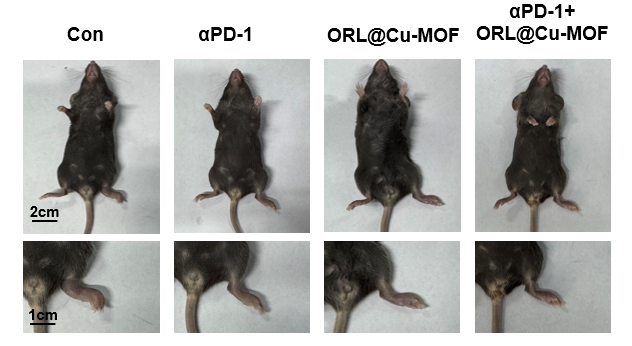


Figure S14. Animal model of popliteal lymph node metastasis induced by injection of MOC-2 cells into foot pads via different treatments.


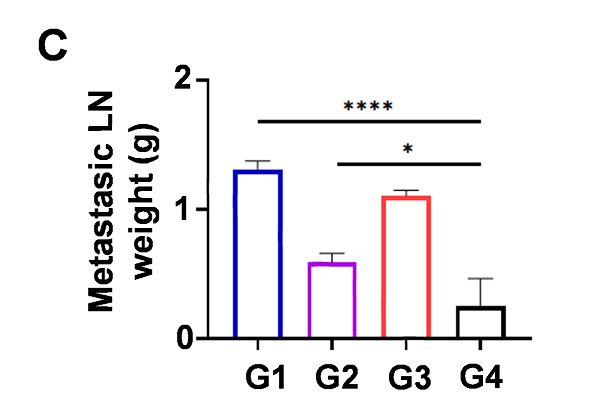


Figure S15. In *vivo* metastatic LN weight with different treatments. G1: Control; G2: αPD-1; G3: ORL@Cu-MOF; G4: αPD-1+ ORL@Cu-MOF. **p* < 0.05, *****p* < 0.0001.


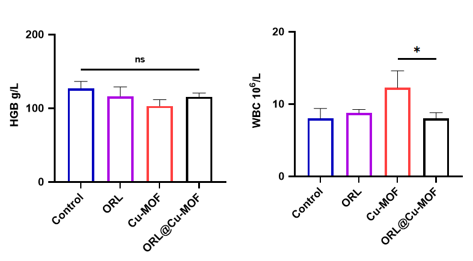


Figure S16. Biochemical analysis of HGB and WBC from mice receiving various treatments. **p* < 0.05, ns, not significant.


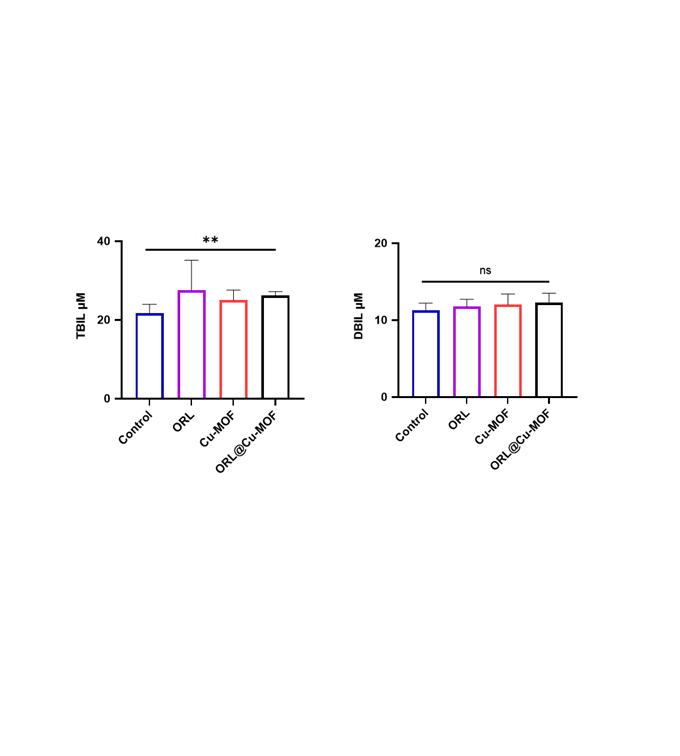


Figure S17. Biochemical analysis of TBIL and DBIL from mice receiving various treatments. ***p* < 0.01.


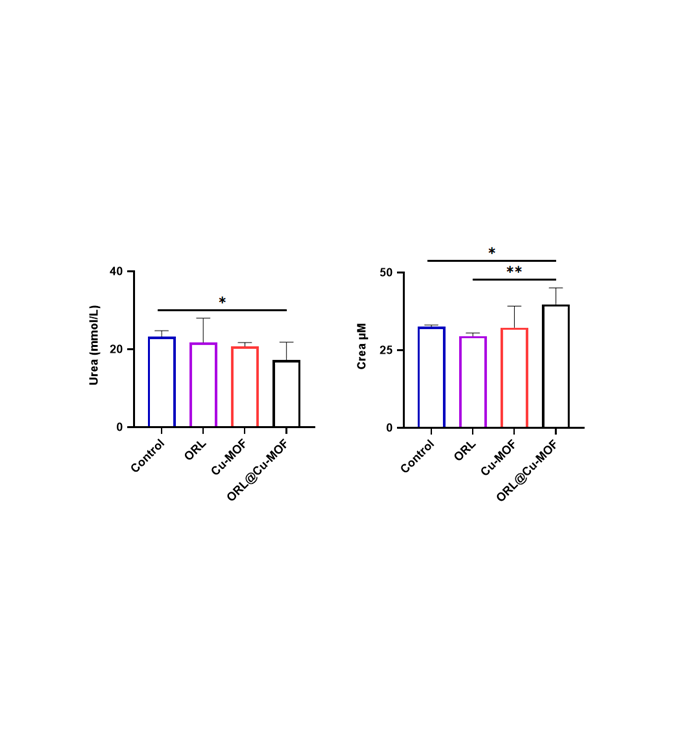


Figure S18. Biochemical analysis of Urea and Crea from mice receiving various treatments. **p* < 0.05, ***p* < 0.01.

**Experimental Section**

**Synthesis**

Copper (II) acetate monohydrate (98%), trimesic acid (98%), and orlistat (98%) were purchased from Shanghai Aladdin Reagent Co., Ltd. N,N-Dimethylformamide (DMF) and ethanol were obtained from Sinopharm Chemical Reagent Co., Ltd. (Shanghai). Copper (II) acetate (0.15 M, 10 mL) and trimesic acid (0.1 M, 10 mL) were simultaneously dripped into an initial solution containing 0.75 mL of DMF and 0.25 mL of ethanol. The dripping process was conducted at a stirring speed of 500 rpm and a rate of 10 mL/h under a temperature of 45°C. After completion of the dripping, the reaction was allowed to proceed for an additional 15 minutes. The product was then centrifuged and washed three times with a mixture of DMF and ethanol to obtain Cu-MOF. The synthesized Cu-MOF was dispersed in 20 mL of ethanol, and 20 mg of orlistat was added. The mixture was stirred overnight in the dark at room temperature. Afterward, the product was centrifuged and washed three times with ethanol to obtain ORL@Cu-MOF.

**Drug Release**

Ten milligrams of ORL@Cu-MOF were dispersed into 10 mL of buffer solutions with pH values of 7.4, 6.8 and 5.5, respectively, and stirred. Every 2 hours, 500 µL of the solution was taken for subsequent testing. The content of Cu ions was measured using inductively coupled plasma optical emission spectrometry (ICP-OES). The ORL content was analyzed using high-performance liquid chromatography (HPLC) with a UV detector set at 205 nm to monitor absorption.

**TMB Assay**

ORL@Cu-MOF at concentrations of 0, 10, 20, 30, 40, and 50 µg/mL were dispersed in an acidic environment with a pH of 5.5. One millimolar of hydrogen peroxide and 5 millimolar of 3,3',5,5'-tetramethylbenzidine (TMB) were added, and the reaction was allowed to proceed for 15 minutes. The UV absorption peaks were then measured in the range of 500-800 nm.

**EPR Test**

Ten milligrams of ORL@Cu-MOF were dispersed into 10 mL of buffer solutions with pH values of 7.4 and 5.5, respectively. 5,5-Dimethyl-1-pyrroline N-oxide (DMPO) was added as a detection reagent to test for the production of ·OH radicals.

**Cell Culture**

The human OSCC cell line SCC-9 and Cal-27 (American Type Culture Collection, Manassas, VA, USA) and mouse oral carcinoma cell line MOC-2 (obtained from Dr. Ravindra Uppaluri, Dana Farber Cancer Institute, Boston, MA) were cultured in DMEM (No. 1196044, Gibco, USA) at 37 ℃ in a humidified atmosphere containing 5% CO_2_. The culture medium was supplemented with 10% fetal bovine serum (SA101, CellMax, China). Cell culture vessels were purchased from NEST (Wuxi, China).

**Antibody**

Rabbit monoclonal DLAT, FDX1, FABP5, ACOX1 and FASN antibody were purchased from ABclonal (A8814, A9815, A0947, A8091 and A0461). Rabbit monoclonal GAPDH and Tubulin antibody were purchased from Abcam (ab9485 and ab18207). Rabbit monoclonal PD-1 and LIAS antibody were purchased from Proteintech (18106-1-AP and 11577-1-AP). Goat anti rabbit HRP binding secondary antibody was obtained from Servicebio (gb23303). Anti-CD11c-PE (PE-65130), anti-CD80-FITC (FITC-65076), anti-F4/80 (98236), anti-CD206 (98031), anti-CD86-APC (APC-65068), anti-CD3-PE (PE-65133) purchased from Proteintech. Anti-CD4-APC and anti-CD8-FITC (A24720 and A23904) were purchased from ABclonal.

**Western Blot Analysis**

Cells were collected and total protein was extracted using RIPA lysis buffer (Beyotime Institute of Biotechnology). Protein concentration was determined using the BCA method. Total protein (10 µg/lane) was separated on a 10% gel using SDS‑PAGE and the separated proteins were transferred to a PVDF membrane (MilliporeSigma). After blocking with 5% fat‑free milk powder in Tris‑buffered saline with 0.05% Tween‑20 (TBST) for 1 h at room temperature, the membrane was incubated with primary antibody at 37˚C for 45 min. After washing with TBST, the membrane was sealed with 5% fat‑free milk powder overnight at 4˚C and incubated with HRP‑bound secondary antibody (1:1,000) in the dark for 1 h at 25˚C. Finally, the protein bands were visualized using the Common ECL chemiluminescence detection kit (cat. no. PK10001; ProteinTech Group, Inc.). ImageJ was used to quantify the western blotting bands, assess the gray values of the different bands, list the gray values obtained and determine the protein expression levels using histograms.

**Cytotoxicity assay**

The Cell Counting Kit‑8 (CCK‑8; Thermo Fisher Scientific, Inc.) assay was used to assess the cytotoxicity of Cal-27, SCC-9 and HaCaT cells. In brief, cells were seeded into 96‑well plates at a density of 3x10^3^ cells/well. Firstly, for the concentration gradient experiment, 0, 10, 20, 30, 40, 50, 60, and 70 μg/ml ORL@Cu-MOF were added to the pores, respectively, add 10 µl of CCK-8 reagent to each well after 12 hours. Secondly, for the cytotoxicity experiment, add 50 μg/ml of ORL@Cu-MOF to each well and add 10 µl of CCK-8 reagent to each well after 12 hours; For HaCaT cells, add 50 μg/ml of ORL@Cu-MOF and then 10 µl of CCK-8 reagent to each well after 12 hours; Cell cytotoxicity was determined by assessing the optical density of each well at a wavelength of 450 nm using a microplate reader (Thermo Fisher Scientific, Inc.).

**Transwell Invasion and Migration Assay**

The ability of cells to invade the Matrigel-coated membrane filter was measured in a transwell chamber assay. The transwell chambers (Merck Millipore Corp., Darmstadt, Germany) were inserted into a 24-well plate and coated with 0.4 mg/mL Matrigel. The cells in the serum-free medium with or without various concentrations of 13-butoxyberberine bromide were cultured in the upper chambers. The lower chamber was filled with 10% FBS medium, which served as a chemoattractant, and incubated for 24 h. After incubation, the non-invaded cells were removed using a cotton swab and the invaded cells on the lower surface of the membrane were fixed with methanol and stained with 0.5% crystal violet. The cells were photographed, and the cell invasion percentage was calculated as compared to the untreated control (0.5% DMSO). For the migration assay, the cells were treated the same as the invasion assay, but without membrane-coating with Matrigel.

**Apoptosis analysis**

Cellular apoptosis was assessed with an Annexin V-FITC apoptosis detection kit (C1062, Beyotime). In brief, Cal-27 cells were seeded on 12-well plates at 3×10^5^ cells per well. Then cells were treated with PBS, ORL, Cu-MOF, ORL@Cu-MOF for 12 h, washed with PBS, and incubated with Annexin/PI reagent in the dark for 15 min at 25°C. Thereafter, the cells were immediately measured with FCM. Moreover, Cal-27 cells were seeded on 12-well plates at 3×105 cells per well. Then cells were treated with PBS, ORL, Cu-MOF, ORL@Cu-MOF for 12 h. After being washed with cold PBS, the spheroids were stained with Calcein AM/PI Cell Viability Kit (C2015, Beyotime). Subsequently, images were collected with CLSM. Data were quantified by ImageJ software.

**Measurement of reactive oxygen species (ROS) production**

Intracellular ROS level was investigated by using dichlorodihydrofluorescein diacetate (DCFH-DA) as a fluorescent probe. In brief, a cover slide was placed in the bottom of each well of a 24-well plate. Cal-27 cells (1×10^5^) in 1 mL complete media were added to each well and incubated at 37°C for 16 h. Afterward, the cells were treated with PBS, ORL, Cu-MOF and ORL@Cu-MOF for 12 h, respectively. The group of cells without any treatment were performed as negative control. Subsequently, the culture medium of the cells was replaced with a serum-free medium and then incubated with ROS indicator DCFH-DA (10 μM) for 20 mins. Subsequently, images were collected with CLSM. Furthermore, the intracellular ROS level was quantified by ImageJ.

**Observation of mitochondrial morphology**

Cal-27 cells were seeded into 6-well plates at a density of 1×106 per well for 12 h. Subsequently, the cells were treated with PBS or ORL@Cu-MOF for 12 h. Then, the cells were collected, fixed by electron microscope fixative (G1102, Servicebio) and observed by Bio-TEM.

**Nile red staining**

For cell lipid droplet quantification, Nile red staining was used. Transfected Cal-27 cells were seeded in 24-well plates in which a sterile glass coverslip had been placed in advance. When the cell layer was between 60% and 80% confluent, the cells were fixed in 2 ml 2% formaldehyde in PBS for 20 min. The cells were then incubated with 1 ml Nile red staining solution (C2051, Beyotime) for 20 min and then stained with Hoechst 33342 (C2051, Beyotime) for 20 min at room temperature. All images were photographed and analysed by using a Leica DM6B microscope.

**Quantification of free fatty acid**

Quantification of fatty acids (FAs) was performed by using an EnzyChrom Free Fatty Acid Assay Kit (BioAssay Systems). All assays were performed following the manufacturer's instructions.

**Animal model**

All animal experimental procedures were performed in accordance with the guidelines provided by the National Institutes of Health Guide for the Care and Use of Laboratory Animals. C57/BL6 mice (female, 4 weeks old) were purchased from Animal Experiment Center of Huazhong Agricultural University and raised in SPF animal rooms. All animal experiments reported herein were performed under the guidelines evaluated and approved by the Committee of Animal Experimentation and the Ethics Committee of School of Stomatology, Wuhan University (Approval number: 50792403002). The mice were housed in accordance with animal welfare regulations, under specific‑pathogen‑free conditions at 25˚C, 50% humidity and a 12‑h light/dark cycle. The animals also had free access to food and water. MOC-2 cells (1x10^5^ cells/100 µl PBS) were injected into the footpad of mice to induce popliteal lymph node metastasis model. At 5, 7, 9 11 and 13 days, they were treated with different drugs via the tail vein. The health and behavior of the animals were monitored every 2 days. Metastatic LN mass and volume (V) were checked. The metastatic LN was almost spherical with a radius of L and the following formula was used: V=4/3πL^3^. The metastatic LN diameter was assessed every 2 days and the metastatic LN volume was determined according to the formula. Subsequently, 14 days following cell injection, the mice were sacrificed via cervical dislocation. The humane endpoints were as follows: A marked reduction in food or water intake, labored breathing, the inability to stand and no response to external stimuli. No abnormal signs that signified the humane endpoints of the experiment were observed in any of the mice during the experiment. When it was confirmed that the experimental animals had no heartbeat or breathing, the metastatic LNs were isolated and weighed.

**Histopathological analysis**

The solid tumor was harvested from tumor-bearing mice on the 14th day of tumor inoculation for histological observation by standard H&E staining. For H&E staining, the excised metastatic LN and organs were fixed in 4% paraformaldehyde solution, embedded in paraffin, sectioned, and stained with H&E. The sections were then observed under a fluorescence microscope (IX83, Olympus).

**Immunohistochemistry**

Formalin-fixed and paraffin-embedded tumor tissues were used for immunohistochemistry. Briefly, following deparaffinization, rehydration, and antigen retrieval, primary antibodies were applied to slides, incubated at 4 °C overnight, and followed with secondary antibody incubation at 37 °C for 30 min. Staining was carried out with 3,3′-diaminobenzidine (DAB), and counter-staining was performed with hematoxylin. The histochemistry score is based on the percentage of respectively positively stained area (1 ≤ 10 %, 2 = 11–30 %, 3 = 31–60 %, 4 = 61–100 %). The histochemistry H score is based on the percentage of positively stained area and staining intensity (no color, light yellow, light brown and brown, graded as 1, 2, 3 and 4, respectively). Calculate the percentage of positive stained area for each grade as positive area/total area × 100 % and add each grade by multiplying the corresponding area.

**Immunofluorescence assay**

The section slices were placed at 65 °C for 4–6 h. The slices were immediately immersed in xylene solution for 10 min and repeated 3 times. After hydration, the slices were put into a buffer containing sodium citrate (1:100) for antigen retrieval. Then, 50-100 μl peroxidase blocker is added to each slice. Goat serum was added on the surface of the sliced tissue for 1 h. After that, diluted primary antibody solution was dropped and incubated overnight at 4 °C. The next day, the primary antibody solution was discarded and sections washed. Corresponding fluorescent secondary antibody solution was dropped to the slices, and incubated in the dark for 1h at room temperature. After washing the slices, DAPI buffer was added. After discarding the DAPI and washing the slices in the dark, the anti-fade agent was dropped. The staining was observed and recorded by OLYMPUS BX51 (Japan).

**Flow cytometric analysis**

After 14 days of treatment, the metastatic LN tissues and TDLN were digested, and dissociated into single cell suspensions. The samples were then resuspended and blocked with 0.1% BSA in PBS, and stained with the corresponding antibody prepared in 0.1% BSA in PBS for 1 h at room temperature. Finally, the cells were detected by FCM.

**Analysis cytokines levels by ELISA assay**

Metastatic LN tissues were collected and homogenized on day of 14th after the last administration. Levels of cytokines in metastatic LN tissues were also detected by ELISA kits according to the protocols, including TNF-α (CME0004, 4A Biotech), IFN-γ (CME0003, 4A Biotech), IL-6 (CME0006, 4A Biotech), IL-12 (CME0013, 4A Biotech).

**Bioinformatics analysis**

Gene Expression Profiling Interactive Analysis (GEPIA, http://gepia.cancer-pku.cn/) was used to analyze the data in The Cancer Genome Atlas (TCGA, https://www.cancer.gov/about-nci/organization/ccg/research/structural-genomics/tcga) to determine the mRNA levels of DLAT, LIAS, FDX1, FASN and the effects of DLAT and FASN on the survival rates of patients with OSCC. Moreover, correlations of DLAT, FASN and PD-1 were analyzed. The expression of DLAT, LIAS, FDX1, FASN in several types of cancers was analyzed on the web of The Human Protein Atlas (https://www.proteinatlas.org/).

**In *vivo* biodistribution study**

The tumor-bearing mice were intravenously injected with Cy7.5 labeled ORL@Cu-MOF (ORL@Cu-MOF@Cy7.5). For the ex *vivo* biodistribution study, mice were sacrificed at 24 h post-injection. Ex vivo imaging of organs including heart, liver, spleen, lung, kidney, intestine, LN and tumor were collected and quantitative analyses using IVIS Spectrum imaging system.

**In *vivo* biosafety evaluation**

Healthy female C57/BL6 mice were randomly grouped (n = 4 mice per group). Each mouse was treated through tail vain, respectively. The mice were monitored and weighed on alternate days. Then the mice were sacrificed at 14 days after administration, and the blood samples of the mice were collected for serum biochemical analyses. Blood sample of each group was centrifuged at 3000 rpm for 10 min to collect plasma, which was further used for analysis of blood biomarkers. Meanwhile, the main organs were also dissected from the mice for further analysis after being sacrificed. All the tissues were paraffin embedded and tissue sections were prepared. Hematoxylin and eosin (H&E) staining was performed on the tissue sessions to observe pathological features, and images were captured using a fluorescence microscope (IX83, Olympus).

**Statistical analysis**

All statistical analyses were performed using GraphPad Prism 8.0 software. Two-tailed unpaired t-tests were used to analyze the two groups. One-way analysis of variance was used to analyze multiplegroups. All results are presented as mean ± SD. The post-hoc test which was used following ANOVA is Tukey’s test. Statistical significance was set at *P*< 0.05.
